# Characterization of Polycyclic Aromatic Hydrocarbons in a Shale Strata Profile from the First Member of the Upper Cretaceous Qingshankou Formation in the Sanzhao Sag, Songliao Basin, NE China

**DOI:** 10.1021/acsomega.4c10835

**Published:** 2025-02-13

**Authors:** Fei Xiao, Jianguo Yang, Yulai Yao, Shichao Li, Yiming Huang, Xiaoyong Gao

**Affiliations:** † Shenyang Center of China Geological Survey/Northeast Geological S&T Innovation Center of China Geological Survey, Shenyang 110034, Liaoning Province, China; ‡ Shale Oil Technology Innovation Center of China Geological Survey, Shenyang 110034, Liaoning Province, China

## Abstract

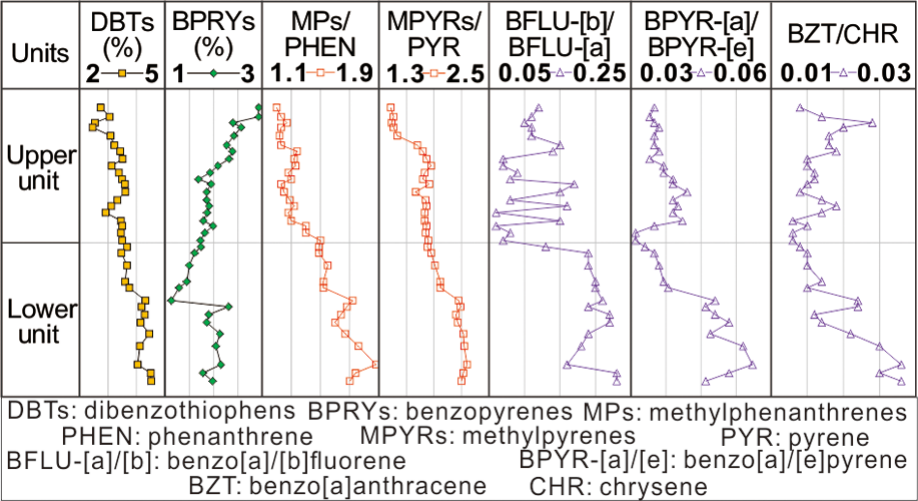

The first member of the Upper Cretaceous Qingshankou Formation (K_2_*qn*^1^) stands as the most significant source rock layer in the Songliao Basin, concurrently serving as the principal target for shale oil exploration. Polycyclic aromatic hydrocarbons (PAHs), as one of the main components of soluble organic matter in mudstone and shale, are of significant importance for revealing the hydrocarbon generation mechanisms in source rocks and the formation conditions of shale oil. However, systematic research on PAHs in the K_2_*qn*^1^ layer of the Songliao Basin has not yet been conducted. Our study concentrated on a comprehensive set of 34 rock core samples, covering the entire K_2_*qn*^1^ layer, retrieved from the SYY3 well in the Sanzhao Sag of the northern Songliao Basin. The geochemical characteristics of a diverse range of PAHs were meticulously assessed through gas chromatography–mass spectrometry (GC–MS). Meanwhile, this study preliminarily discussed possible influential factors on the formation of alkylated PAHs (*a*-PAHs) and the isomerization of parent PAHs (*p*-PAHs) in our samples. The results revealed that PAHs predominantly consist of the phenanthrene, naphthalene, and chrysene series, trailed by the pyrene, fluorene, dibenzothiophene, and benzopyrene series. In contrast to the lower unit (>2015.00 m) of the K_2_*qn*^1^ layer, the upper unit (<2015.00 m) exhibits generally lower PAH concentrations and reduced levels of dibenzothiophene series, implying lower biological productivity and more oxidized sedimentary waters. The upper unit exhibits a higher content of 1,2,5-trimethylnaphthalene, 1,2,5,6-tetramethylnaphthalene, retene, pyrenes, fluoranthene, benzopyrenes, and benzofluoranthene, suggesting elevated levels of contributions from terrestrial higher plants. Maturity parameters of alkyl naphthalene and methylphenanthrene, along with vitrinite reflectance (*R*_o_), indicate a close maturity in both units. Most of the *a*-PAHs/*p*-PAH ratios are higher in the lower unit than in the upper unit, indicating more pronounced alkylation. The ratios of *p*-PAH isomers, including benzo[b]fluorene/benzo[a]fluorene, benzo[a]pyrene/benzo[e]pyrene, and benzo[a]anthracene/chrysene, exhibit a vertical distribution pattern similar to the *a*-PAHs/*p*-PAH ratios, indicating that less stable *p*-PAH isomers are more prevalent in the lower unit. By comparing the *a*-PAHs/*p*-PAH ratios and the ratios of *p*-PAH isomers with conventional geochemical parameters of saturated hydrocarbons, it was preliminarily revealed that the catalytic effects of clay minerals, along with fluctuating biological inputs, can substantially affect PAH alkylation and *p*-PAH isomerization. Sediment reductivity slightly enhances PAH alkylation without obviously impacting *p*-PAH isomerization, and salinity shows no significant effect on these processes. The above insights offer molecular geochemical evidence of PAHs, which aids in understanding the heterogeneity of the K_2_*qn*^1^ source rock, facilitates oil source correlation, and optimizes the selection of sweet spots within shale oil formations.

## 1 Introduction

Aromatic hydrocarbons are prevalent components in crude oil and the soluble organic matter of oil shale, coal, and other fossil fuels. In crude oils that have not been subjected to secondary alterations, such as biodegradation or water washing, aromatic hydrocarbons typically constitute a proportion that is second only to that of saturated hydrocarbons, yet exceeds that of nonhydrocarbons and asphaltenes.^1−3^ They account for about 10–30% of the total crude oil and 10–45% of the total hydrocarbon content.^4−6^ The combined use of column chromatography and gas chromatography–mass spectrometry (GC–MS) constitutes a potent analytical technique for examining the aromatic fractions in crude oil and the soluble organic matter of mudstone and shale. These fractions mainly consist of primary polycyclic aromatic hydrocarbons (PAHs) with two or more benzene rings, their alkyl derivatives with varying carbon numbers, and certain heteroatomic aromatic hydrocarbons containing sulfur or oxygen. GC–MS is capable of detecting common heteroatomic aromatic hydrocarbons, including dibenzothiophene, dibenzofuran, and their alkyl derivatives, in crude oils and source rocks originating from diverse geological backgrounds. These compounds share the fundamental molecular structure of condensed aromatics, which comprises two benzene rings and a five-membered heterocyclic ring. In this article, we studied polycyclic aromatic hydrocarbons (PAHs), which include both purely aromatic and heteroatomic aromatic species, as present in the aromatic fraction isolated by column chromatography. PAHs in crude oil and source rocks are commonly classified into several categories: bicyclic (the naphthalene and biphenyl series), tricyclic (the phenanthrene, anthracene, dibenzothiophene, dibenzofuran, fluorene series), tetracyclic (the chrysene, pyrene, and triaromatic steroid series, fluoranthene), and pentacyclic (the perylene series, benzopyrene, benzofluoranthene) aromatic hydrocarbons.^7−16^ Additionally, hexacyclic (indene[cd]pyrene, benzo[ghi]perylene) and heptacyclic (halobenzene) aromatic hydrocarbons can be detected in crude oil and source rocks with low content;^17^ however, they are usually found in higher contents in coal.^18^

PAHs in geological strata can originate from the incomplete combustion of biomass, diagenetic alterations of natural biolipids, and molecular liberation during the thermal maturation of kerogen.^18−20^ In petroleum geochemistry, PAHs are utilized to discern the biological origins of organic matter. While the biological sources of certain PAHs, such as the phenanthrene and chrysene series, remain elusive, specific markers such as 1,2,5-trimethylnaphthalene, 1,2,7-trimethylnaphthalene, and cadalene (4-isopropyl-1,6-dimethylnaphthalene) are indicative of the biological source from terrigenous higher plants. Triaromatic steroids were thought to be derived from the aromatization of sterols, sterones, and steric acids from planktonic algae.^7,21−23^ However, the sedimentary environment also exerts an influence on the distribution of PAHs.^5^ For instance, the pronounced presence of benzothiophene and dibenzothiophene is suggestive of a carbonate or evaporite environment.^24^ The distribution characteristics of monoarylsteranes and triaromatic steroids can differentiate between continental and marine crude oils and can be employed to estimate water salinity.^2,23,25^ The relative composition of dibenzothiophene, dibenzofuran, and fluorene can reflect the redox conditions of the sedimentary environment.^26,27^ The sedimentary facies and lithology can be inferred from the cross-plot of the ratios of dibenzothiophene to phenanthrene (DBT/PHEN) and pristane to phytane (Pr/Ph).^28^ PAHs exhibit a broader range of chemical kinetics compared to saturated hydrocarbons, which gives them a unique advantage in assessing the thermal evolution of organic matter with higher maturity. Many maturity parameters related to alkylnaphthalenes, alkylphenanthrenes, and alkyl dibenzothiophenes have been proposed and are extensively applied to evaluate the thermal evolution degree of crude oils and source rocks.^21,22,24,29−38^ Moreover, PAHs can be utilized to investigate the biodegradation effects on crude oil, the fractionation effects of oil migration, and so forth, providing an effective approach to comprehend the oil and gas accumulation processes.^6,39−42^

The above applications of PAHs capitalize on the intrinsic structural variations among different biological precursor PAHs or the alterations in the PAH molecular structures that take place throughout geological processes. The processes of PAH alkylation and isomerization, which are regulated by specific mechanisms, have become significant areas of interest in organic geochemistry. There is broad consensus that the extent of PAH alkylation is correlated with thermal maturity.^43−46^ It has been demonstrated that the degree of alkylation is maturity-dependent under conditions of similar lithologies and organic matter types. Furthermore, alkylation indices of the phenanthrene series can be utilized to evaluate the thermal maturity of highly mature to overmature samples.^47^ An inverse relationship was noted between the generation temperature of crude oil and the degree of PAH alkylation, with high-temperature-generated oils showing low alkylation and low-temperature-generated oils showing high alkylation.^48^ Furthermore, it was indicated that the lithology of source rocks and the type of kerogen significantly influence these processes.^48,49^ Marine crude oils generally have higher relative contents of alkylated PAHs compared to their parent compounds, with the degree of alkylation differing based on the source rock lithology—carbonate-derived oils show the highest, paralic-derived oils the lowest, and siliciclastic-derived oils are intermediate.^48^ It was demonstrated that in the Gippsland Basin of Australia, Cretaceous-Paleogene source rocks contain dibenzofurans and their C_0_-C_4_ alkyl-substituted derivatives. These compounds are primarily controlled by the source of organic matter, not by thermal maturity or the depositional environment. Furthermore, the ratio of methyldibenzofurans to dibenzofuran may serve as a paleoclimatic indicator and an oil-source correlation tool.^49^ PAH isomerization, akin to PAH alkylation, is also influenced by multiple factors including maturity, lithology, and mineral composition, and it plays a pivotal role in assessing the maturity parameters of commonly used alkylnaphthalenes, alkylphenanthrenes, and alkyldibenzothiophenes.^45^ Lithology can notably affect the isomerization process, potentially resulting in anomalous aromatic maturity parameter values.^45,50,51^ Studies indicate that clay minerals catalyze the isomerization of the alkylphenanthrene series, and that methylphenanthrene isomerization may be moderated by sulfide minerals.^45,52^ In summary, on the one hand, existing research on the alkylation of PAHs has primarily focused on the impact of thermal maturity, with relatively limited attention given to nonmaturity factors, such as diverse depositional environments and sources of organic matter. On the other hand, while there has been considerable research on the isomerization of alkylated PAHs (*a*-PAHs), there is a scarcity of studies on the parent PAHs (*p*-PAHs).

**Figure 1:**
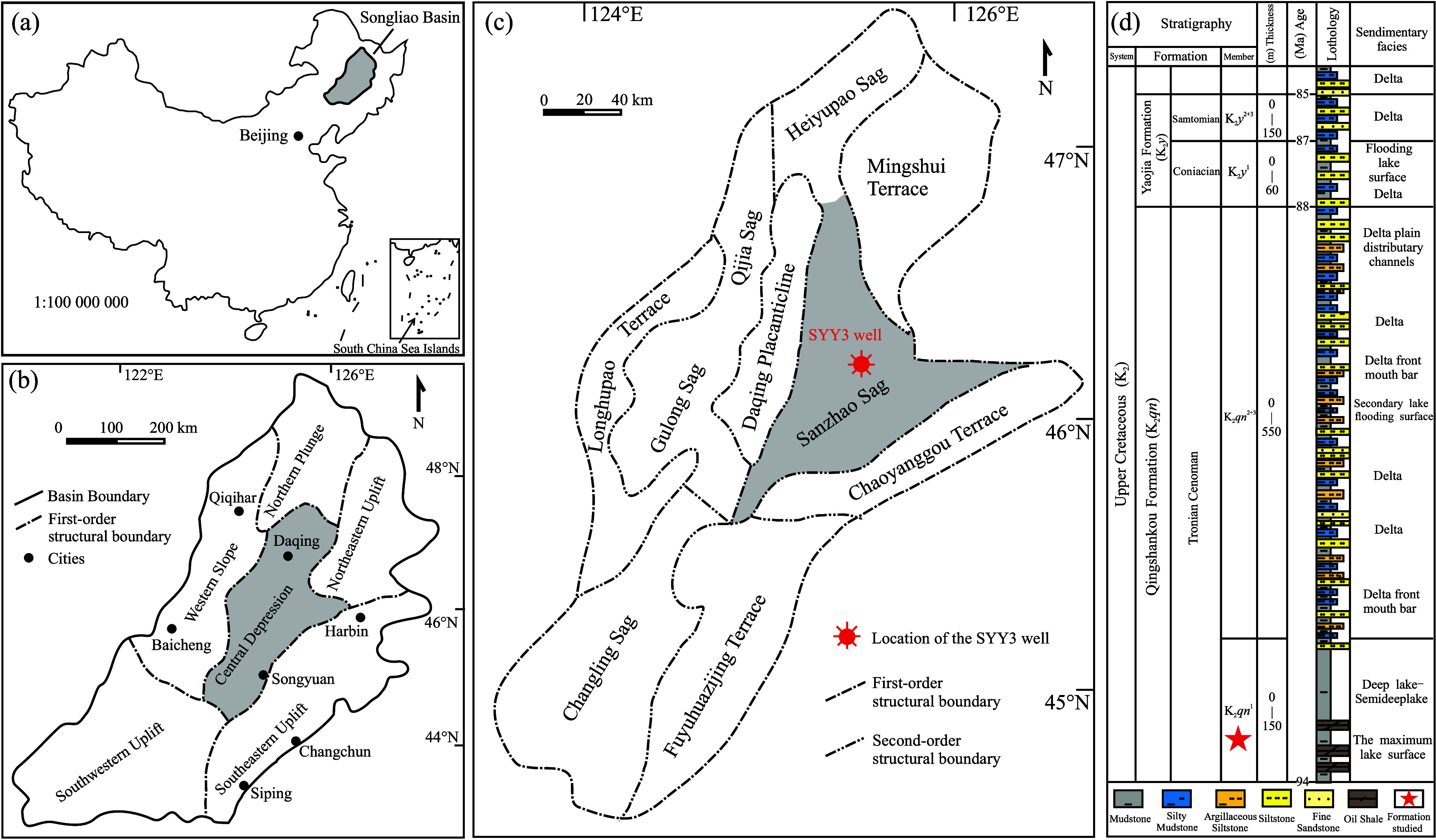
Geological and structural overview of the Songliao Basin: (a) location of the basin in China; (b) first-order structural units of the basin; (c) second-order structural units in the Central Depression of this basin, with the study area and the SYY3 well highlighted; and (d) stratigraphic column showing the K_2_*qn*^1^ layer. This figure was adapted from the reference of Yang et al.^60^ Reprinted (Adapted or Reprinted in part) with permission from [Yang, J.; Wang, L.; Li, S.; Zuo, C.; Xiao, F.; Chen, Y.; Yao, Y.; Bai, L. The influence of reservoir composition on the pore structure of continental shale: A case study from the Qingshankou Formation in the Sanzhao Sag of the Northern Songliao Basin, NE China. Geofluids 2021, 2021, 5869911. 10.1155/2021/5869911.] Copyright [2021] [Wiley].

In this study, we examined the Sanzhao Sag of the Central Depression in the Songliao Basin, NE China (Figure 1a–c), focusing on the primary hydrocarbon source rock layer to address issues in the aforementioned PAHs research. The Songliao Basin, one of the largest continental oil-bearing basins in China, features the first member of the Upper Cretaceous Qingshankou Formation (K_2_*qn*^1^) shale strata (Figure 1d) as a pivotal source rock and the main target for shale oil exploration.^53−57^ These source rocks are predominantly located in the Qijia, Gulong, Sanzhao, and Changling hydrocarbon-generating sags (Figure 1c).^58−61^ Prior geochemical studies have characterized PAHs in crude oils and source rocks from the Songliao Basin. A systematic investigation of PAH distribution and composition in northern Songliao Basin crude oils identified a total of 11 series, including naphthalenes, phenanthrenes, chrysenes, biphenyls, fluorenes, dibenzofurans, dibenzothiophenes, and triaromatic steroids, comprising 164 aromatic compounds.^4^ Crude oils from the Qijia and Gulong sags were categorized into three groups based on the triaromatic steroid series content in PAHs: high (40–57%), low (4.24–13.52%), and trace (0.45–0.76%). The characteristics of crude oils containing triaromatic steroids and low ratios of DBT/PHEN (0.002–0.032) suggest that the source rocks formed in a weakly oxidizing, freshwater-to-brackish water environment.^4^ In the southern Songliao Basin, the naphthalene series predominates among PAHs in crude oils, followed by the phenanthrene and biphenyl series, indicating deposition in a weakly oxidizing, weakly reducing freshwater lacustrine environment with significant terrigenous higher plant input.^11^ Maturity criteria for alkylnaphthalenes, methylphenanthrenes, and triaromatic steroids were applied to assess whether the K_2_*qn*^1^ samples from the southern Songliao Basin were within the oil generation window.^62,63^ The relative contents of dibenzothiophene, dibenzofuran, and fluorene series indicated a strongly reducing sedimentary environment for the K_2_*qn*^1^ layer in the Changling Sag.^64^ However, comprehensive studies on the geochemical properties of PAHs within the K_2_*qn*^1^ shale strata have not been conducted, and reports on the alkylation and isomerization of PAHs are scarce.

The published paper has utilized the SYY3 well, the first shale oil parameter well in the Sanzhao Sag, as a case study to conduct a comprehensive investigation into the geochemical characteristics of saturated hydrocarbons in the K_2_*qn*^1^ samples.^65^ The findings indicate that the K_2_*qn*^1^ shale strata were deposited in a reducing, brackish lacustrine setting, where prokaryotes were the main biological source of organic matter with supplementary input from eukaryotic algae. For the SYY3 well, the K_2_*qn*^1^ layer was stratified into the upper and lower units, delineated by a boundary at a depth of 2015.00 m. The lower unit, in contrast to the upper, was characterized by more pronounced reducing conditions, a higher content of clay minerals, and reduced salinity, with a more significant biogenic contribution from eukaryotic algae than from prokaryotes.^65^ In this study, we performed a detailed analysis of the composition and distribution of PAHs in the K_2_*qn*^1^ samples from the SYY3 well, integrating the outcomes of the molecular geochemistry of saturated hydrocarbons. This analysis was aimed at elucidating the potential significance of these PAHs in relation to the biogenic precursors of hydrocarbon generation and the sedimentary environment. Subsequently, we investigated the alkylation patterns of PAHs and the isomerization trends of *p*-PAHs within the K_2_*qn*^1^ stratigraphic sequence and delved into the principal factors that exert control over these processes.

## 2 Geological Setting

The Songliao Basin, located in northeastern China, is an extensive Mesozoic-Cenozoic continental sedimentary basin that covers an area of approximately 26 × 10^4^ km^2^ (Figure 1a). The basin’s evolution can be divided into five stages: early folding, initial tensile fracturing, cleavage, subsidence, and atrophy-equilibrium.^66^ The basin consists of six primary tectonic units: the Central Depression, the Northern Plunge, the Western Slope, the Southwestern Uplift, the Northeastern Uplift, and the Southeastern Uplift (Figure 1b). The Central Depression, encompassing ten secondary structural units including the Qijia, Gulong, Sanzhao, Changling, and Heiyupao sags, the Longhupao, Mingshui, Chaoyanggou, and Fuyuhuazijing terraces, and the Daqing Placanticline (Figure 1c), contains the majority of the basin’s oil and gas resources. The basin features thick clastic sediments from the Jurassic to the Neogene in its center, with a thickness of about 10 km.^67^ During the subsidence phase, two prolific sets of source rocks, the Upper Cretaceous Qingshankou and Nenjiang formations, developed in the Songliao Basin. The primary source rock for oil is the Qingshankou Formation, which consists of the first (K_2_*qn*^1^), second, and third (K_2_*qn*^2+3^) members, particularly the deep to semi-deep lacustrine dark mudstone and shale of the K_2_*qn*^1^ layer (Figure 1d). The K_2_*qn*^1^ layer is highly prospective for oil generation due to its high organic matter abundance, oil-prone kerogen, and medium to high maturity, making it a significant target for shale oil exploration.^53^

The Sanzhao Sag, situated in the mid-eastern part of the Central Depression in the Songliao Basin (Figure 1c), spans an area of 5743 km^2^. The tight sandstone reservoir of the fourth member of the Lower Cretaceous Quantou Formation (K_1_*q*^4^) has been the traditional focus for petroleum exploration and development in the Sanzhao Sag, with the K_2_*qn*^1^ source rock serving as the primary oil source.^68,69^ In recent years, the Sanzhao Sag’s K_2_*qn*^1^ shale oil has garnered increasing attention following significant breakthroughs in shale oil geological surveys in the Qijia and Gulong sags. Shenyang Center of the China Geological Survey, in collaboration with Petrochina Daqing Oilfield, has drilled the SYY3 well—the first shale oil parameter well in this sag—located in the central part of the study area in 2019 (Figure 1c). The K_2_*qn*^1^ mudstone and shale reservoir in this well has demonstrated promising oil and gas shows, as evidenced by the seepage of yellow-green shale oil from core fissures. Small-scale hydraulic fracturing and oil testing have yielded a total industrial oil flow rate of 3.46 m^3^/day.^55^ The K_2_*qn*^1^ interval ranges in depth from 1968.50 to 2061.50 m with a thickness of 93.00 m, while the SYY3 well reaches a drilling depth of 2547.00 m. The entire K_2_*qn*^1^ layer was cored during drilling, providing a rock core sample base for this study.

## 3 Samples and Methods

### 3.1 Sampling and Previous Data

The thirty-four core samples utilized in this study were retrieved from the SYY3 well in the Sanzhao Sag (Figure 1c), covering the entire K_2_*qn*^1^ layer. The lithological compositions of these samples are primarily mudstone and shale, with a minority consisting of silty mudstone and oil shale (Table 1). During the sampling process, efforts were made to ensure that the samples were evenly distributed in depth and that the sampling density was appropriately increased based on lithological variations, to guarantee a representative reflection of the K_2_*qn*^1^ layer. The published paper offers a wealth of data on total organic carbon, rock pyrolysis, and the column chromatography separation of group components from the above thirty-four core samples, as well as gas chromatography (GC) and GC–MS analyses of the saturated hydrocarbons present in these samples.^65^ In this study, the geochemical data of PAHs were evaluated through GC–MS analysis of the aromatic fraction extracted from the soluble organic matter in the rock samples.

**Table 1:** Geochemical Data of Group Components and Added Anthracene-*d*_10_ Standard Volumes for the K_2_*qn*^1^ Samples in the SYY3 Well^a^

depth (m)	lithology	mass of group components (mg)				standard volumes (μL)	percentage of group components (%)^b^				ARO/(SAT + ARO) (%)
		saturates	aromatics	nonhydrocarbons	asphaltenes		saturates^b^	aromatics^b^	nonhydrocarbons^b^	asphaltenes^b^	
1971.04	shale	3.90	1.17	1.08	0.02	10	63.18	19.05	17.47	0.30	23.17
1974.15	shale	4.14	1.20	0.89	0.01	10	66.30	19.19	14.30	0.22	22.45
1976.05	mudstone	4.68	1.39	1.09	0.02	10	65.21	19.39	15.13	0.26	22.92
1977.56	silty mudstone	3.16	1.22	1.00	0.03	10	58.47	22.58	18.46	0.49	27.86
1980.30	shale	5.41	2.69	1.41	0.04	10	56.67	28.18	14.75	0.39	33.21
1983.29	mudstone	14.50	4.75	0.40	1.80	20	67.60	22.14	1.86	8.39	24.68
1985.35	mudstone	4.00	1.70	1.30	2.30	10	43.01	18.28	13.98	24.73	29.82
1987.80	mudstone	5.50	1.75	1.00	2.40	10	51.64	16.43	9.39	22.54	24.14
1990.01	silty mudstone	11.00	2.85	1.40	3.40	10	58.98	15.28	7.51	18.23	20.58
1992.25	mudstone	9.00	2.80	1.40	2.60	10	56.96	17.72	8.86	16.46	23.73
1994.33	mudstone	10.85	3.05	0.45	1.20	10	69.77	19.61	2.89	7.72	21.94
1995.96	mudstone	12.90	5.25	0.50	0.50	20	67.36	27.42	2.61	2.61	28.93
1998.55	shale	18.35	11.60	0.50	0.65	30	59.00	37.30	1.61	2.09	38.73
2001.15	shale	28.00	10.00	0.55	0.10	30	72.45	25.87	1.42	0.26	26.32
2003.08	shale	12.50	5.10	2.75	5.85	20	47.71	19.47	10.50	22.33	28.98
2005.23	mudstone	8.30	3.50	1.95	0.45	10	58.45	24.65	13.73	3.17	29.66
2007.90	mudstone	12.10	5.40	2.45	3.80	20	50.95	22.74	10.32	16.00	30.86
2009.53	mudstone	3.55	3.30	1.75	4.30	10	27.52	25.58	13.57	33.33	48.18
2011.79	shale	11.15	5.45	2.35	1.70	20	54.00	26.39	11.38	8.23	32.83
2014.26	mudstone	11.05	3.65	1.70	1.00	10	63.51	20.98	9.77	5.75	24.83
2016.38	mudstone	11.60	5.05	2.00	2.65	20	54.46	23.71	9.39	12.44	30.33
2018.39	mudstone	12.80	4.15	1.70	10.05	20	44.60	14.46	5.92	35.02	24.48
2022.34	mudstone	8.65	4.55	2.00	6.05	20	40.71	21.41	9.41	28.47	34.47
2027.62	shale	10.90	4.30	2.05	8.05	20	43.08	17.00	8.10	31.82	28.29
2029.61	mudstone	12.45	3.50	1.90	7.15	10	49.80	14.00	7.60	28.60	21.94
2033.82	mudstone	9.05	2.45	1.35	4.40	10	52.46	14.20	7.83	25.51	21.30
2035.80	oil shale	4.55	2.65	1.65	0.55	10	48.40	28.19	17.55	5.85	36.81
2038.37	mudstone	12.05	6.30	2.55	2.75	20	50.95	26.64	10.78	11.63	34.33
2040.94	mudstone	13.35	5.40	2.60	3.70	20	53.29	21.56	10.38	14.77	28.80
2044.57	oil shale	5.90	2.70	1.50	1.45	10	51.08	23.38	12.99	12.55	31.40
2048.60	mudstone	8.15	3.00	1.40	2.55	10	53.97	19.87	9.27	16.89	26.91
2054.63	mudstone	3.15	1.80	1.50	1.25	10	40.91	23.38	19.48	16.23	36.36
2057.32	oil shale	7.10	3.00	1.35	2.45	10	51.08	21.58	9.71	17.63	29.70
2060.00	mudstone	10.40	2.65	1.85	8.40	10	44.64	11.37	7.94	36.05	20.31

a Note: ARO/(SAT + ARO) = aromatics × 100/(saturates + aromatics).

b The percentage data of group components were referenced from the reference of Xiao et al.^65^ Reprinted (Adapted or Reprinted in part) with permission from [Xiao, F.; Yang, J.; Li, S.; Yao, Y.; Huang, Y.; Gao, X. Enrichment and movability of lacustrine tight shale oil for the first member of the Upper Cretaceous Qingshankou Formation in the Sanzhao Sag, Songliao Basin, NE China: Insights from saturated hydrocarbon molecules. Fuel 2024, 368, 131615. 10.1016/j.fuel.2024.1316 15.] Copyright [2024] [Elsevier].

### 3.2 GC–MS Analysis

In preparation for GC–MS analysis, the samples underwent a meticulous pretreatment process. This entailed the size reduction of the sample through comminution, extraction of soluble organic compounds, and separation of group components to isolate the aromatic fraction. For a detailed explanation of these methods, please consult the published paper.^65^ Before the samples were subjected to injection, an anthracene-*d*_10_ internal standard, precisely measured at a concentration of 0.1042 μg/μL, was incorporated into each sample. The volume of the standard added varied according to the mass of the aromatic fraction isolated, with selections ranging from 10 to 30 μL to ensure the accuracy of quantification. Specifically, for aromatic fraction contents below 4 mg, 10 μL of the standard was introduced; for contents scaling from 4 to 10 mg, the volume was upped to 20 μL; and for contents surpassing 10 mg, a full 30 μL of the standard was added (Table 1). The aromatic fractions of these samples were evaluated using an Agilent 6890N-5975B system equipped with a DB-5 ms elastic quartz capillary column (60 m × 0.25 mm × 0.25 μm). Helium (99.999% purity) served as the carrier gas with a flow rate of 1.0 mL/min. The temperatures of the injector and the transfer line were set at 290 and 250 °C, respectively, and unsplit stream sampling was employed for injection. The oven temperature was initially set at 70 °C for 2 min, then ramped up to 310 °C at a rate of 3 °C/min, and held at 310 °C for 18 min. The ion source in the mass spectrometer utilized electron ionization (EI) at 70 eV. The scanning modes included both full scan (SCAN) and selective ion monitoring (SIM), with a mass range of 50–600 amu.

Aromatic compounds were mainly identified in accordance with the People’s Republic of China’s national standard, known as the “The test method for biomarkers in sediment and crude oil by GC–MS” (GB/T 18606-2017). Quantitative analysis of the aromatic compounds was conducted using mass chromatograms corresponding to their characteristic ions, with relevant parameters determined based on the peak areas of all detected PAHs. The identification of aromatic hydrocarbons was facilitated by specific mass-to-charge ratios (*m*/*z*) (Figure S1). For the naphthalene series (NAPs), *m*/*z* values of 128, 142, 156, 170, 184, and 198 were utilized (Figure S1a). Phenanthrene series (PHENs) were characterized by *m*/*z* values of 178, 192, 206, 220, and 234 (Figure S1b). Chrysene (CHR) and benzo[a]anthracene (BZT) were identified using *m*/*z* 228, while C_1_–C_2_ alkyl CHRs were indicated by *m*/*z* 242 and 256 (Figure S1c). Benzofluoranthene (BFRT) and benzopyrenes (BPYRs) were marked by *m*/*z* 252 (Figure S1c). Fluoranthene (FRT) and pyrene (PYR) were characterized by *m*/*z* = 202, while benzofluorene series (BFLUs) and methyl PYRs were indicated by an *m*/*z* of 216 (Figure S1d). Fluorene series (FLUs) were represented by *m*/*z* values of 166, 180, and 194 (Figure S2a). Dibenzothiophen series (DBTs) were identified through *m*/*z* 184, 198, 212, and 226 (Figure S2b). Biphenyl (BIP) was detected at *m*/*z* 154, methyl BIPs and dibenzofuran (DBF) at *m*/*z* 168, C_2_ alkyl BIPs and methyl DBFs at *m*/*z* 182, and C_2_ alkyl DBFs at *m*/*z* 196 (Figure S2c). Triaromatic steroid series (TASs) were identified through *m*/*z* values of 231 and 245 (Figure S2d). The PAH concentration results are presented as the mass ratio of the compound to the rock, utilizing the unit of micrograms per gram (μg/g) (Table 2).

**Table 2:** Concentration Data of the Major PAHs of the K_2_*qn*^1^ Samples in the SYY3 Well^a^

depth (m)	lithology	bicyclic aromatic compounds (μg/g)		tricyclic aromatic compounds (μg/g)				tetracyclic aromatic compounds (μg/g)						pentacyclic aromatic compounds (μg/g)	
		NAPs	BIPs	PHENs	FLUs	DBTs	DBFs	CHRs	PYRs	BZT	TASs	BFLUs	FRT	BPYRs	BFRT
1971.04	shale	16.313	0.441	50.766	3.715	1.549	0.004	4.532	1.889	0.024	1.125	0.205	0.089	1.111	0.192
1974.15	shale	18.602	0.447	48.784	2.324	1.528	0.004	4.365	1.770	0.024	1.023	0.214	0.068	1.045	0.200
1976.05	mudstone	17.388	0.445	54.116	3.163	1.638	0.004	4.801	1.831	0.032	1.619	0.240	0.085	1.022	0.217
1977.56	silty mudstone	13.595	0.346	44.269	3.097	1.332	0.004	4.002	1.571	0.024	1.352	0.199	0.074	0.886	0.194
1980.30	shale	27.888	0.646	70.525	2.622	2.221	0.006	6.327	2.445	0.035	1.805	0.337	0.097	1.307	0.288
1983.29	mudstone	15.356	0.430	38.011	3.059	1.750	0.005	5.595	2.298	0.041	1.356	0.266	0.058	1.236	0.222
1985.35	mudstone	6.709	0.186	45.639	2.992	2.248	0.008	7.107	3.005	0.055	1.680	0.308	0.069	1.562	0.283
1987.80	mudstone	7.518	0.207	41.947	3.018	2.137	0.007	6.885	2.803	0.042	1.570	0.286	0.059	1.415	0.270
1990.01	silty mudstone	5.966	0.169	25.879	1.959	1.186	0.003	4.174	1.595	0.025	1.552	0.178	0.035	0.792	0.153
1992.25	mudstone	8.727	0.248	37.165	2.912	1.797	0.005	5.630	2.297	0.036	1.251	0.259	0.046	1.024	0.205
1994.33	mudstone	3.662	0.110	11.553	0.934	0.572	0.001	1.667	0.697	0.010	0.373	0.078	0.019	0.278	0.060
1995.96	mudstone	49.391	1.186	88.580	7.124	4.541	0.011	13.567	5.752	0.076	1.942	0.614	0.109	2.468	0.495
1998.55	shale	22.624	0.562	45.398	3.400	2.282	0.002	6.354	2.846	0.037	0.388	0.297	0.106	1.175	0.242
2001.15	shale	25.353	0.639	41.881	3.459	1.965	0.005	6.202	2.809	0.043	0.432	0.280	0.048	1.099	0.223
2003.08	shale	9.682	0.271	37.372	2.780	1.641	0.004	5.875	2.509	0.043	0.470	0.300	0.049	1.004	0.218
2005.23	mudstone	9.898	0.282	36.099	2.643	1.475	0.003	5.605	2.359	0.038	0.396	0.262	0.049	0.944	0.205
2007.90	mudstone	21.786	0.583	78.406	5.591	3.795	0.008	11.770	5.548	0.064	0.701	0.549	0.103	1.966	0.425
2009.53	mudstone	10.817	0.309	46.100	3.464	2.270	0.003	7.088	3.333	0.044	0.173	0.273	0.053	1.290	0.244
2011.79	shale	13.764	0.411	44.417	3.774	2.150	0.004	6.472	3.072	0.034	0.169	0.296	0.038	1.131	0.209
2014.26	mudstone	7.840	0.226	41.537	3.246	2.012	0.005	5.604	2.856	0.030	0.180	0.250	0.041	0.991	0.179
2016.38	mudstone	15.260	0.429	63.489	4.568	3.214	0.009	8.535	4.458	0.049	0.241	0.332	0.067	1.521	0.267
2018.39	mudstone	10.811	0.329	33.967	2.887	1.627	0.003	4.600	2.157	0.027	0.080	0.205	0.033	0.753	0.133
2022.34	mudstone	11.982	0.344	44.892	3.689	2.270	0.006	6.053	2.912	0.036	0.089	0.265	0.046	0.927	0.158
2027.62	shale	9.943	0.278	19.741	1.774	0.984	0.005	2.651	1.192	0.018	0.062	0.122	0.016	0.396	0.068
2029.61	mudstone	14.092	0.407	29.368	2.711	1.515	0.003	3.721	1.728	0.021	0.086	0.176	0.025	0.522	0.091
2033.82	mudstone	10.838	0.300	24.210	2.226	1.447	0.003	3.223	1.362	0.023	0.094	0.139	0.020	0.379	0.070
2035.80	oil shale	37.572	1.021	104.784	8.790	6.343	0.014	18.286	6.389	0.118	0.885	0.566	0.084	3.495	0.527
2038.37	mudstone	27.870	0.748	63.822	5.766	3.903	0.010	9.949	3.622	0.048	0.526	0.358	0.050	1.726	0.269
2040.94	mudstone	23.682	0.635	38.297	3.751	2.270	0.008	5.953	2.070	0.030	0.393	0.226	0.029	1.020	0.163
2044.57	oil shale	63.496	1.535	152.198	12.830	9.776	0.022	25.608	9.347	0.161	1.342	0.860	0.117	4.669	0.705
2048.60	mudstone	33.164	0.921	128.077	10.420	7.579	0.017	22.548	7.333	0.163	1.283	0.747	0.098	3.781	0.599
2054.63	mudstone	28.951	0.762	168.431	10.552	9.673	0.027	31.097	9.764	0.256	1.104	0.755	0.143	5.155	0.792
2057.32	oil shale	14.322	0.405	55.824	4.910	3.603	0.006	9.496	3.089	0.070	0.314	0.361	0.043	1.428	0.238
2060.00	mudstone	5.642	0.179	21.143	1.826	1.384	0.002	3.930	1.067	0.031	0.040	0.150	0.028	0.605	0.113

a Note: NAPs = naphthalene series; BIPs = biphenyl series; PHENs = phenanthrene series; FLUs = fluorene series; DBTs = dibenzothiophen series; DBFs = dibenzofuran series; CHRs = chrysene series; PYRs = pyrene series; BZT = benzo[a]anthracene; TASs = triaromatic steroid series; BFLUs = benzofluorene series; FRT = fluoranthene; BPYRs = benzopyrene series; and BFRT = benzofluoranthene.

## 4 Results and Discussion

### 4.1 Common Geochemical Characteristics of PAHs

#### 4.1.1 General Composition and Distribution of PAHs

**Figure 2:**
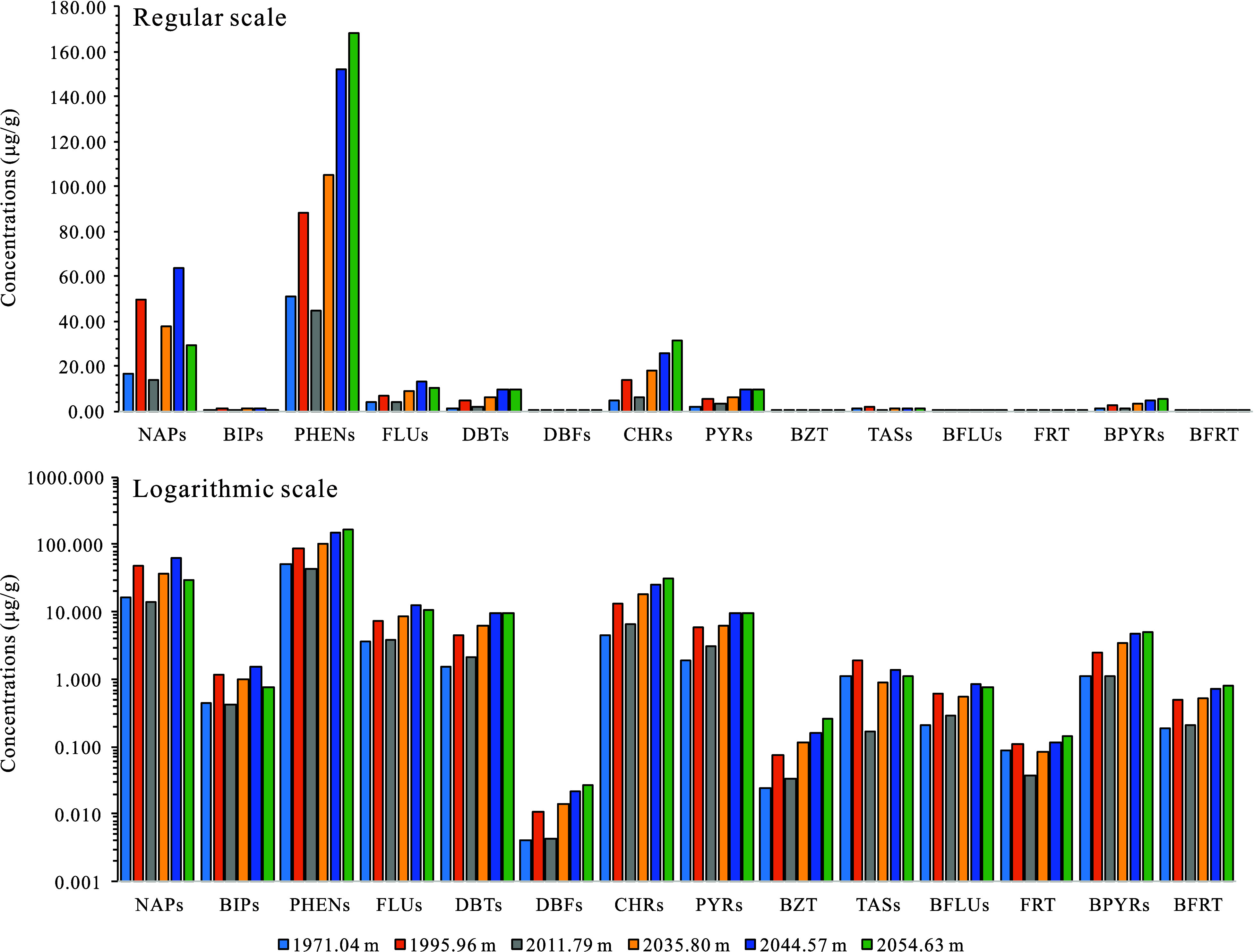
Concentration distribution histogram of PAHs in the K_2_*qn*^1^ samples from the SYY 3 well, Sanzhao Sag (the six sampling points indicated by the colored arrows in Figure 3).

The data from the group component analysis reveal that the soluble organic matter in the K_2_*qn*^1^ layer from the SYY3 well is primarily composed of saturated hydrocarbons, with aromatic hydrocarbons or asphaltenes being the next most abundant components. Nonhydrocarbons are present in comparatively smaller quantities (Table 1). Notably, the proportion of aromatic hydrocarbons varies from 11.37 to 37.30%, with an average of 21.44%, and they account for 20.31 to 48.18% of the total hydrocarbon content (ARO/(SAT + ARO)), averaging at 28.51% (Table 1). These results are comparable to the levels of aromatic hydrocarbons reported in previous studies on Chinese crude oils.^4−6,70^ The PAHs identified in the samples include bicyclic (NAPs, BIPs), tricyclic (PHENs, FLUs, DBTs, and DBFs), tetracyclic (CHRs, PYRs, BZT, TASs, BFLUs, and FRT), and pentacyclic (BPYRs, BFRT) aromatic hydrocarbons (Table 2, Figure 2). PHENs are particularly prevalent, with concentrations varying from 11.553 to 168.431 μg/g and averaging at 55.197 μg/g. NAPs also show significant concentrations, ranging from 3.662 to 63.496 μg/g, with an average of 18.250 μg/g. CHRs is another notable component, present in concentrations between 1.667 and 31.097 μg/g, averaging at 8.214 μg/g. Other PAHs, such as FLUs, DBTs, PYRs, BPYRs, and TASs, are detected in varying concentrations. Conversely, compounds such as BIPs, DBFs, BZT, BFLUs, FRT, and BFRT are found in much lower concentrations, all with average levels below 0.500 μg/g (Table 2). Excluding TASs, the concentrations of various PAHs in the lower unit of the K_2_*qn*^1^ layer are generally higher compared to the upper unit (Table 2, Figure 2). This pattern aligns with the greater organic matter enrichment observed in the lower unit.^65^ The observation suggests that the lower unit experienced a period of enhanced biological productivity during deposition, surpassing that of the upper unit.

**Figure 3:**
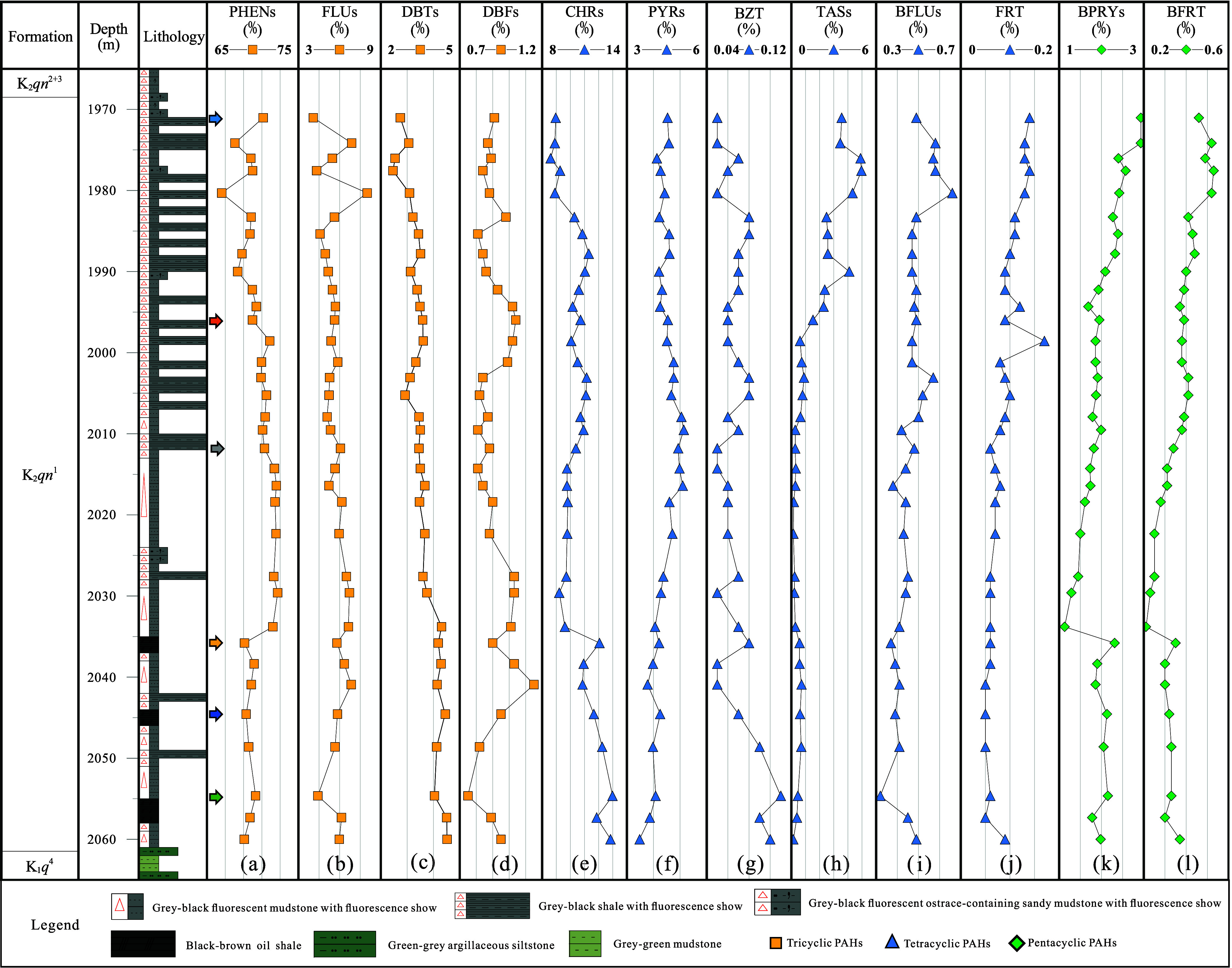
Comprehensive geochemical profile of PAHs in the K_2_*qn*^1^ samples from the SYY 3 well, Sanzhao Sag: relative percentage contents of (a) PHENs; (b) FLUs; (c) DBFs; (d) DBTs; (e) CHRs; (f) PYRs; (g) BZT; (h) TASs; (i) BFLUs; (j) FRT; (k) BPRYs; and (l) BFRT. NAPs and BIPs were excluded from the total PAHs in calculating the aforementioned values.

**Figure 4:**
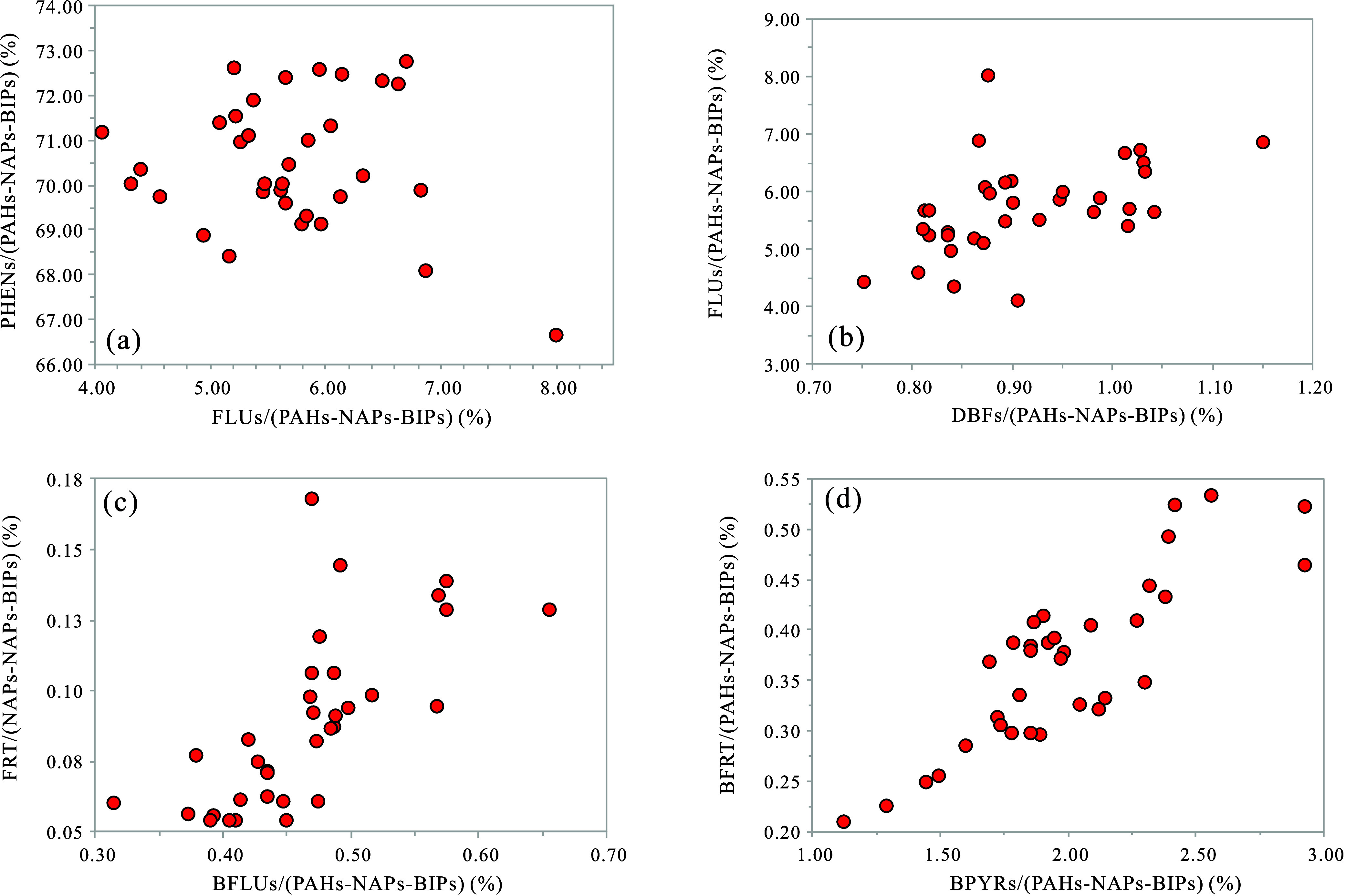
Comparative percentage content plots for the K_2_*qn*^1^ samples from the SYY 3 well, Sanzhao Sag: (a) PHENs/(PAHs-NAPs-BIPs) vs FLUs/(PAHs-NAPs-BIPs); (b) FLUs/(PAHs-NAPs-BIPs) vs DBFs/(PAHs-NAPs-BIPs); (c) FRT/(PAHs-NAPs-BIPs) vs BFLUs/(PAHs-NAPs-BIPs); and (d) BFRT/(PAHs-NAPs-BIPs) vs BPYRs/(PAHs-NAPs-BIPs).

It is important to note that the concentrations of naphthalenes and biphenyls, which are more volatile with fewer rings, do not accurately represent their original levels due to potential losses of light components during sample preparation. Thus, we compared the relative percentage contents of PAHs in the K_2_*qn*^1^ layer, excluding NAPs and BIPs. The distribution patterns of several PAHs within the K_2_*qn*^1^ layer are shown in Figure 3 and Table S1. The relative content of PHENs was between 65 and 75% across the profile, with lower values at the top and bottom and higher contents in the middle and upper parts (Figure 3a). There is a slight negative correlation between the relative contents of PHENs and FLUs (Figures 3a,b and 4a), suggesting that geological factors influencing these compounds may be, to some extent, antagonistic. The relative content of DBTs consistently increases with burial depth (Figure 3c), indicating a more reducing sedimentary environment from top to bottom. The relative content of DBFs shows a notable positive correlation with that of FLUs (Figure 4b), implying a probable genetic connection due to their similar molecular structures.^67^ The relative contents of BFLUs, FRT, BPRYs, and BFRT all generally decrease with an increase in depth (Figure 3i–l). Furthermore, there is a strong positive correlation between the relative contents of BFLUs and FRT (Figure 4c), as well as between BPRYs and BFRT (Figure 4d). This suggests a common origin for BFLUs, FRT, BPRYs, and BFRT, and it indicates differences in the sources of organic matter with depth.

#### 4.1.2 Biotic Input of Organic Matter Identified from PAHs

**Figure 5:**
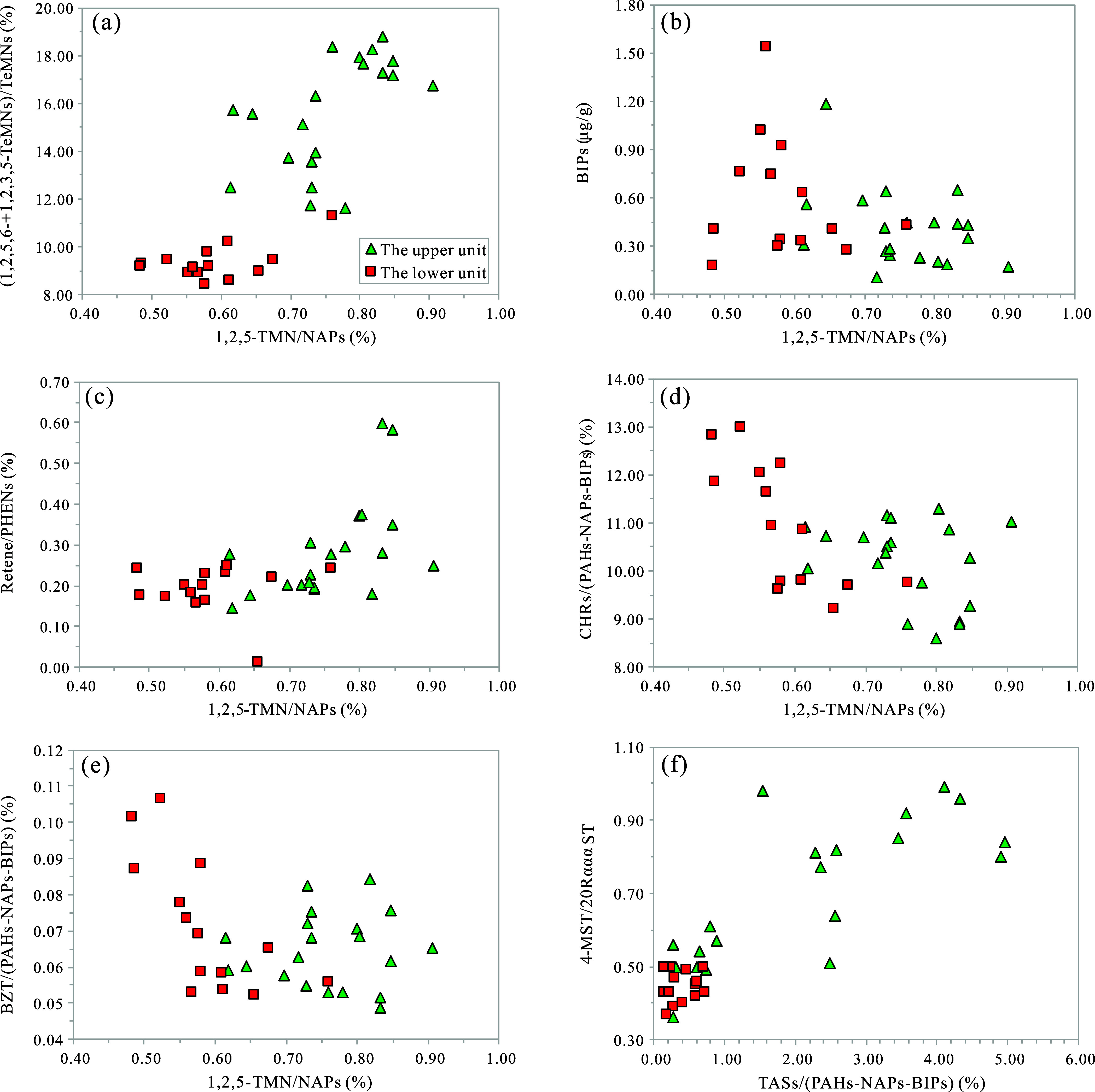
Correlative analysis plots of typical saturated and aromatic geochemical parameters indicating the source of biotic input in the K_2_*qn*^1^ samples from the SYY 3 well, Sanzhao Sag: (a) (1,2,5,6 + 1,2,3,5-TeMN)/TeMNs vs 1,2,5-TMN/NAPs; (b) concentration of BIPs vs 1,2,5-TMN/NAPs; (c) Retene/PHENs vs 1,2,5-TMN/NAPs; (d) CHRs/(PAHs-NAPs-BIPs) vs 1,2,5-TMN/NAPs; (e) BZT/(PAHs-NAPs-BIPs) vs 1,2,5-TMN/NAPs; and (f) 4-MST/20RαααST vs TASs/(PAHs-NAPs-BIPs). TeMN(s) = tetramethylnaphthalene (series); TMN = trimethylnaphthalene; 4-MST = 4-methylstane steranes; and 20RαααST = C_27–29_ 20Rααα steranes.

Although the biological origins of most PAHs are complex, certain specific PAHs can be traced back to the biogenic input of organic matter in the source rocks. Alkyl-substituted NAPs, due to their molecular structural similarities with natural products, were believed to originate from sesquiterpenoid to triterpenoid molecules in higher plants and microbes.^22^ The biogenic significance of terrigenous higher plants is particularly associated with 1,2,5-trimethylnaphthalene (1,2,5-TMN), 1,2,7-trimethylnaphthalene (1,2,7-TMN), and 1,2,5,6-tetramethylnaphthalene (1,2,5,6-TeMN). 1,2,7-TMN was thought to form from the oleanane molecular skeleton in angiosperms after the Cretaceous period, while 1,2,5-TMN and 1,2,5,6-TeMN can be derived from the pentacyclic triterpene vanillin in higher plants or the bicyclic diterpenoid hypopericylic acid from resins.^8,21^ The absence of 1,2,7-TMN in the soluble organic matter of the K_2_*qn*^1^ samples suggests that there was no significant biogenic input from angiosperms after the Cretaceous period, aligning with the Late Cretaceous depositional background of these samples. Table S2 and Figure 5a illustrate that in the upper unit, the percentage content of 1,2,5-TMN in NAPs (1,2,5-TMN/NAPs) varies from 1.98 to 5.86%, averaging 3.61%, and the combined percentage content of 1,2,5,6-TeMN and 1,2,3,5-TeMN in TeMNs ((1,2,5,6 + 1,2,3,5-TeMN)/TeMNs) ranges from 11.62 to 18.77%, averaging 15.61%. In contrast, in the lower unit, the 1,2,5-TMN/NAPs ratio varies from 1.67 to 3.07%, averaging 2.36%, and the (1,2,5,6 + 1,2,3,5-TeMN)/TeMNs ratio ranges from 8.43 to 11.32%, averaging 9.35%. Consequently, the upper unit generally contains higher contents of 1,2,5-TMN, and likely 1,2,5,6-TeMN, suggesting a greater input of terrestrial higher plant biogenic material in this sublayer.

Although BIPs and retene are present in low contents in our samples (Tables 2 and S2), their unique implications for biotic input make them worthy of some discussion. BIPs were believed to originate from the lignin of higher plants.^7,71,72^ Additionally, BIPs can be produced through the coupling process of phenolic compounds.^73^ However, some research indicates that BIPs do not have clear biological precursors.^74^ In the upper unit, BIP concentrations range from 0.110 to 1.186 μg/g, with an average of 0.407 μg/g. In the lower unit, BIP concentrations range from 0.179 to 1.535 μg/g with an average of 0.592 μg/g (Table 2). There is a weak negative correlation between BIP concentrations and the 1,2,5-TMN/NAPs ratio (Figure 5b). These observations suggest the differences in biotic input between the upper and lower units. Furthermore, the primary source of BIPs in this study may not be terrestrial higher plants. Retene (1-methyl-7-isopropyl phenanthrene), one of the most studied PHENs (Figure S1b), was traditionally viewed as a diagenetic product derived from biological precursors during the diagenetic process after sedimentation, specifically from abietic acid. Abietic acid, a common diterpenoid acid found in conifer (coniferae) resins, has led to the consideration of retene as a marker for coniferous forest plant resins in geological history.^75,76^ However, the origin of retene was understood to be more diverse in other studies, not only from higher plants but also from algae and bacteria.^77−79^ The percentage content of retene relative to phenanthrene (Retene/PHENs) in the upper unit ranges from 0.15 to 0.60%, averaging at 0.28%, whereas in the lower unit, it ranges from 0.01 to 0.25%, averaging at 0.19% (Table S2 and Figure 5c). Additionally, the Retene/PHENs ratio shows a positive correlation with the 1,2,5-TMN/NAPs ratio (Figure 5c). These findings indicate that retene in the samples likely originates from terrestrial higher plants, and the comparatively higher Retene/PHENs ratio in the upper unit suggests a greater biogenic input from terrestrial higher plants in this sublayer.

**Figure 6:**
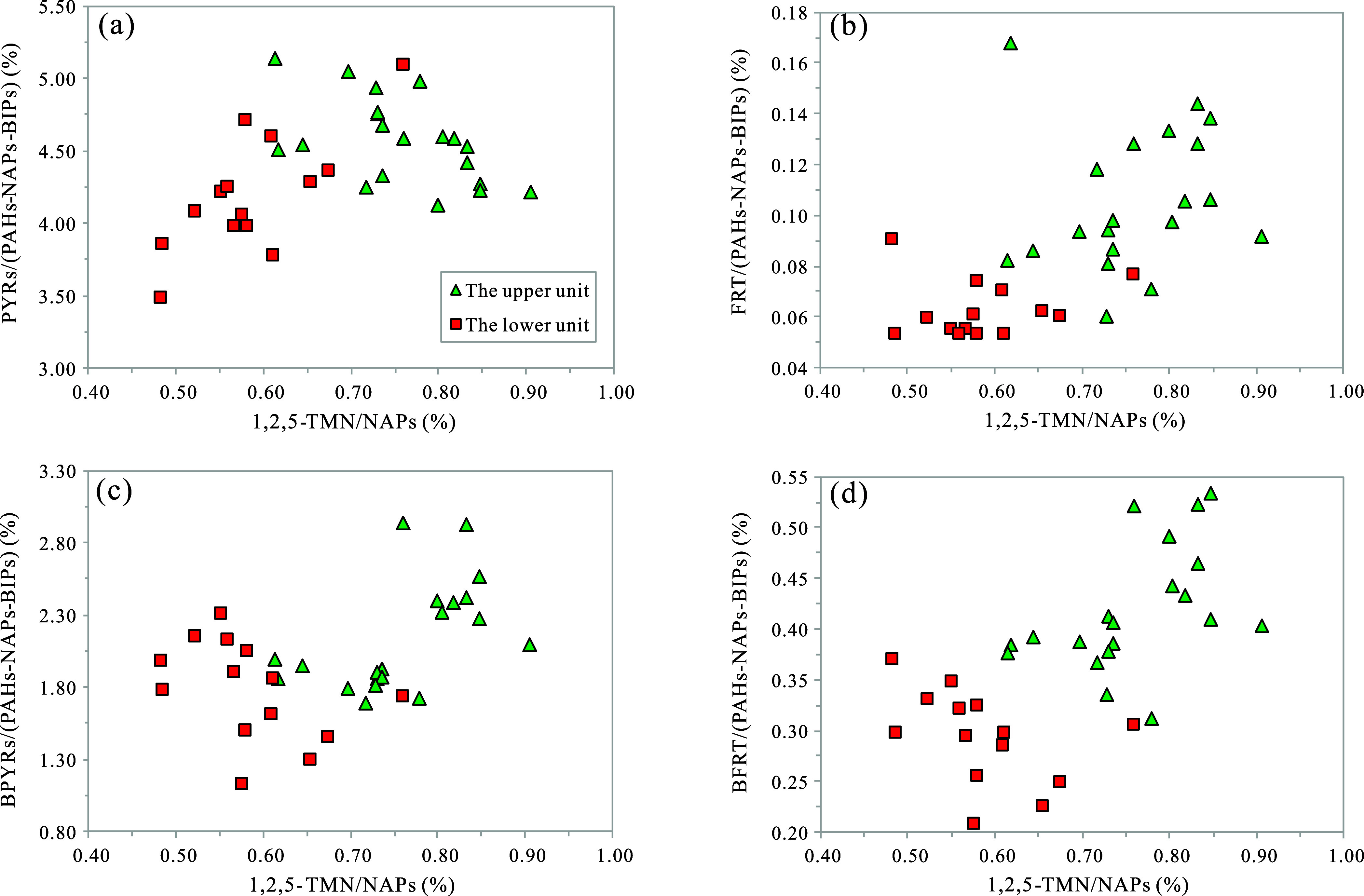
Correlative analysis plots of typical aromatic geochemical parameters indicating the source of biotic input in the K_2_*qn*^1^ samples from the SYY 3 well, Sanzhao Sag: (a) PYRs/(PAHs-NAPs-BIPs) vs 1,2,5-TMN/NAPs; (b) FRT/(PAHs-NAPs-BIPs) vs 1,2,5-TMN/NAPs; (c) BPYRs/(PAHs-NAPs-BIPs) vs 1,2,5-TMN/NAPs; and (d) BFRT/(PAHs-NAPs-BIPs) vs 1,2,5-TMN/NAPs.

CHRs, as common PAHs, currently have undefined implications for biotic input, with some studies suggesting that CHRs may originate from lower aquatic organisms.^72,80^ That can be supported to some degree by the relative content of CHRs exhibiting a negative correlation with 1,2,5-TMN/NAPs in our samples (Figure 5d). The relative contents of CHRs are slightly higher in the lower unit compared with the upper unit (Figures 3e and 5d). This difference implies a marginally higher biogenic input from lower aquatic organisms in the lower unit. BZT, an isomer of CHR, is seldom discussed in the literature in relation to its biogenic origins. According to our data, BZT, much like CHRs, shows a negative correlation with the 1,2,5-TMN/NAPs ratio, and the lower unit exhibits a higher BZT content than the upper unit (Figures 3g and 5e). This suggests that BZT may originate from the same biological sources as CHRs. A positive correlation exists between the 1,2,5-TMN/NAPs ratio and the percentage contents of PYRs, FRT, BPYRs, and BFRT, with the upper unit exhibiting higher contents of these PAHs than the lower unit (Figure 6). This suggests that these PAHs likely originate from terrestrial higher plants, consistent with previous study findings,^11,13^ and indicates a greater contribution from higher terrigenous plants in the upper unit. CHRs, FRT, and PYR can be formed from biological precursor molecules during diagenesis, but they can also result from biomass burning.^81−83^ The ratios FRT/(FRT + PYR) and BZT/(BZT + CHR) were suggested to reflect the relative contributions of combustion and petrogenic sources to these PAHs. Values of FRT/(FRT + PYR) < 0.40 and BZT/(BZT + CHR) < 0.20 imply a petrogenic (petroleum) source.^84,85^ For the K_2_*qn*^1^ layer in the SYY3 well, the FRT/(FRT + PYR) ratio ranges from 0.013 to 0.027, averaging 0.018, and the BZT/(BZT + CHR) ratio ranges from 0.033 to 0.091, averaging 0.053 (Table S2). These ratios suggest a petrogenic origin for CHR, BZT, FRT, and PYR in the K_2_*qn*^1^ layer.

TASs, resembling a sterane carbon skeleton, are typically considered to be the aromatization products of monoarylsteranes that result from heating at depth.^23^ A positive correlation exists between the percentage content of TASs and the ratio of 4-methylsteranes to C_27–29_ 20Rααα steranes (4-MST/20RαααST) (Figure 5f). This correlation suggests that TASs and 4-methylsteranes share a similar biogenetic origin, primarily from prokaryotes such as bacteria and acritarch.^65^ This differs from the previous view that triaromatic steranes originate from planktonic algae.^7,21−23^ Furthermore, the upper unit exhibits a higher percentage contents of TASs and a higher 4-MST/20RαααST ratio compared with the lower unit (Figure 5f). This indicates a greater prokaryotic biogenic contribution to the organic matter in the upper unit.

In summary, the upper unit exhibits elevated levels of specific PAHs, including 1,2,5-TMN, 1,2,5,6-TeMN, BIPs, Retene, PYRs, FRT, BPYRs, and BFRT, which are suggestive of substantial biogenic input from terrestrial higher plants. In the saturated hydrocarbon biomarker compounds, the ratios of C_19–20_ tricyclic terpanes to C_21–26_ tricyclic terpanes and C_24_ tetracyclic terpanes to C_26_ tricyclic terpanes in the upper unit of the K_2_*qn*^1^ layer from the SYY3 well are significantly higher than those in the lower unit, also verifying a higher terrigenous higher plant input characteristic in the relatively shallow aquatic environment of the upper unit.^65^ Furthermore, the enhanced presence of TASs points toward a significant biogenic contribution by prokaryotes in the upper unit. In contrast, the decreased levels of certain PAHs, such as CHRs and BZT, indicate a reduced biogenic input from lower aquatic organisms, consistent with results from the lower ratios of tricyclic terpanes to pentacyclic terpanes and C_27_ 20Rααα sterane to C_29_ 20Rααα sterane in the upper unit.^65^

#### 4.1.3 Palaeosedimentary Environment Identified from PAHs

**Figure 7:**
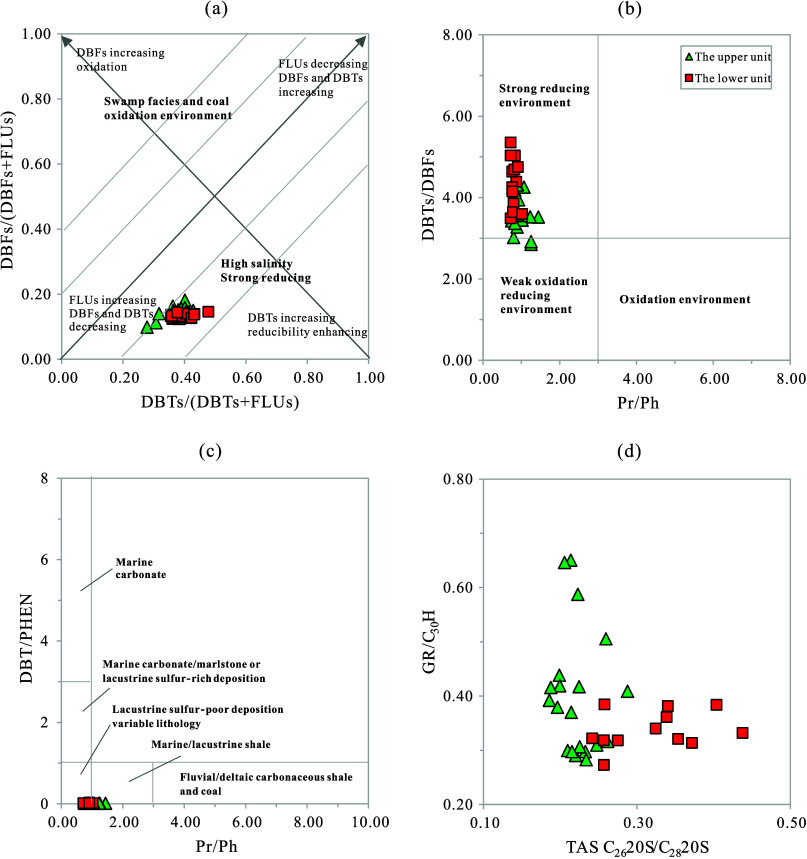
Geochemical ratio plots for the K_2_*qn*^1^ samples from the SYY 3 well, Sanzhao Sag: (a) DBFs/(DBFs+FLUs) vs DBTs/(DBTs+FLUs); (b) DBTs/DBFs vs Pr/Ph; (c) DBT/PHEN vs Pr/Ph; and (d) GR/C_30_H vs TAS C_26_20S/C_28_20S. Pr = pristine; Ph = phytane; GR = gammacerane; C_30_H = C_30_ hopane.

It was previously believed that DBTs, FLUs, and DBFs share a common precursor molecule with the chemically active C-9 carbon atom of the five-membered ring being a key site for transformation (Figure S1b). This atom can be modified under various redox conditions: it can be sulfurized to form DBTs in strongly reducing environments, hydrogenated to form FLUs in reducing environments, or oxygenated to form DBFs in weakly oxidizing and weakly reducing environments. Consequently, the relative proportions of DBTs, FLUs, and DBFs can reflect the redox characteristics of the sedimentary environment.^70^ However, simulation experiments have suggested that these three series of PAHs may originate from different precursors. FLUs can be produced from β-carotene through demethylation and transmethylation during aromatization, while DBTs and DBFs may originate from biphenyl.^74^ In light of these findings, the plots of DBFs/(DBFs + FLUs) vs DBTs/(DBTs + FLUs) and DBTs/DBFs vs pristane/phytane (Pr/Ph) were proposed to indicate the nature of the sedimentary environment.^26^ The data from the K_2_*qn*^1^ layer of the SYY3 well suggest deposition in a reducing to strongly reductive environment with some salinity, with the lower unit experiencing more reductive conditions than the upper unit (Figure 7a,b). Specifically, the DBFs/(DBFs + FLUs) ratios for the upper and lower units are 0.10–0.18 (averaging 0.14) and 0.13–0.15 (averaging 0.14), respectively; the DBTs/(DBTs + FLUs) ratios are 0.28–0.43 (averaging 0.37) and 0.36–0.48 (averaging 0.40), respectively; the DBTs/DBFs ratios are 2.85–4.31 (averaging 3.62) and 3.49–5.36 (averaging 4.36), respectively; the Pr/Ph ratios are 0.75–1.44 (averaging 0.97) and 0.72–1.02 (averaging 0.80), respectively (Table S2). Therefore, the differences in these sedimentary environmental parameters are not significant, which is related to the fact that the K_2_*qn*^1^ layer in the SYY3 well is generally characterized by a deepwater-reducing semi-deep lake to a deep lake environment.

The plot of DBT/PHEN versus Pr/Ph serves as a practical tool in our research, effectively indicating the sedimentary environment and lithology of the source rocks. The DBT/PHEN ratio, as a lithology indicator, typically exceeds 1.0 in carbonate rocks and is less than 1.0 in shales. Meanwhile, the Pr/Ph ratio reflects the redox conditions of the sedimentary environment. By integrating these parameters, we can discern various sedimentary settings and lithologies.^2,28^ Our data from the SYY3 well (Figure 7c) show that samples from the upper unit are distributed across two areas: marine/lacustrine shale and lacustrine sulfur-poor deposition. In contrast, samples from the lower unit are predominantly found in the lacustrine sulfur-poor deposition area. This distribution corresponds to the geological observation that lacustrine shale interbeds are more developed in the upper unit of the SYY3 well, whereas they are less developed in the lower unit (Figure 3). From the differences in geochemical parameters, the DBT/PHEN ratios for the upper and lower units are 0.011–0.018 (averaging 0.015) and 0.015–0.021 (averaging 0.018), respectively (Table S2), with the lower unit showing a slightly higher value. Considering the slightly lower Pr/Ph ratio in the lower unit (Table S2), it can be determined that the lower unit was deposited in a sedimentary environment that was slightly deeper, more reducing, and slightly sulfur-rich.

The distribution characteristics of TASs, influenced by the salinity of sedimentary waters, are crucial for understanding sedimentary environments. A previous study of crude oils with different genetic types in the Jiyang Depression, Bohai Bay Basin, eastern China, showed that C_28_ TASs were more prevalent in organic matter formed in freshwater environments, while C_26_ TASs were more abundant in organic matter formed in brackish to saline-water environments.^5^ Consequently, the ratio of C_26_ TAS 20S to C_28_ TAS 20S (TAS C_26_20S/C_28_20S) serves as an effective indicator for distinguishing biogenic input and sedimentary environments, with saline-water lacustrine crude oils characterized by TAS C_26_20S/C_28_20S ratios greater than 0.45.^5^ Our data from the K_2_*qn*^1^ layer of the SYY3 well (Table S2 and Figure 7d) show a TAS C_26_20S/C_28_20S range from 0.19 to 0.44, with an average of 0.26, suggesting the presence of brackish water bodies. Specifically, the C_26_20S/C_28_20S ratios for the upper and lower units are 0.19–0.29 (averaging 0.22) and 0.24–0.44 (averaging 0.32), respectively; the GR/C_30_H ratios are 0.28–0.65 (averaging 0.40) and 0.27–0.52 (averaging 0.35), respectively (Table S2). Interestingly, these two parameters indicate opposite salinity results for the water bodies; the C_26_20S/C_28_20S ratios suggest higher salinity in the lower water body, while the GR/C_30_H ratios indicate higher salinity in the upper water body. We speculate that this contradiction may be related to the possible marine incursion that the K_2_*qn*^1^ in the Sanzhao Sag experienced,^65^ which complicates the interpretation of the salinity in the sedimentary environment’s water body. Marine incursion events were reported to occur in the upper of the K_2_*qn*^1^ layer, leading to an abnormal increase of salinity in some layers.^65^ Therefore, we consider that the GR/C_30_H ratios can be more reliable parameters to indicate the salinity of water bodies for our samples.

### 4.2 Differential Distribution of p-PAH and the Corresponding a-PAHs

#### 4.2.1 Profile Variation of PAH Alkylation

**Figure 8:**
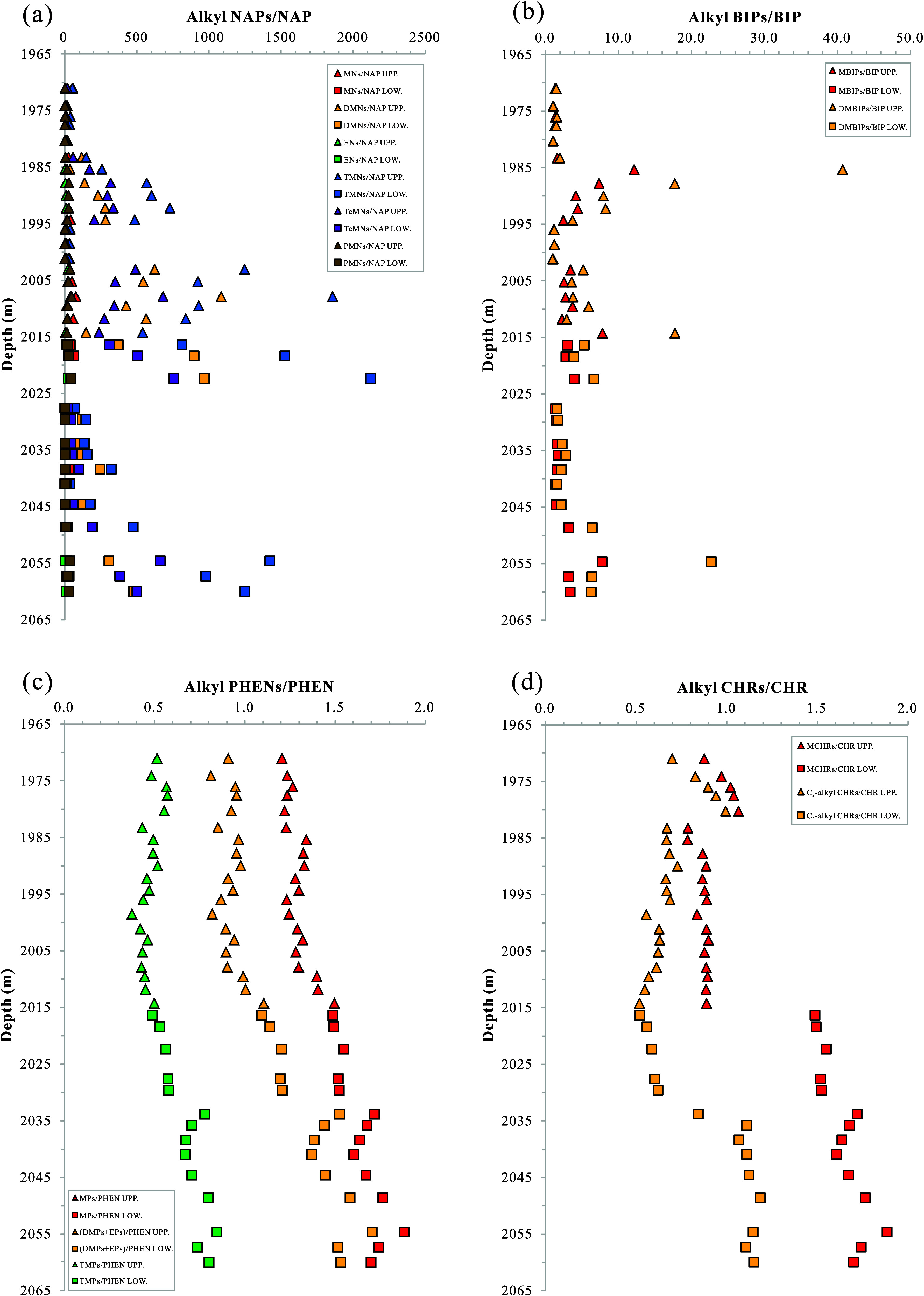
Variation diagrams of content ratios of *a*-PAHs to *p*-PAH of (a) NAPs, (b) BIPs, (c) PHENs, and (d) CHRs with buried depth of the K_2_*qn*^1^ samples from the SYY3 well in the Sanzhao Sag. UPP. = in the upper unit; LOW. = in the lower unit; NAP = naphthalene; MNs = methylnaphthalene series; DMNs = dimethylnaphthalene series; ENs = ethylnaphthalene series; TMNs = trimethylnaphthalene series; TeMNs = tetramethylnaphthalene series; PMNs = pentamethylnaphthalene series; BIP = biphenyl; MBIPs = methylbiphenyl series; DMBIPs = dimethylbiphenyl series; PHEN = phenanthrene; MPs = methylphenanthrene series; DMPs = dimethylphenanthrene series; EPs = ethylphenanthrene series; TMPs = trimethylphenanthrene series; CHR = chrysene; MCHRs = methylchrysene series; C_2_-alkyl CHRs = C_2_ alkylchrysene series.

Each class of PAHs consists of *p*-PAH and their alkyl derivatives, known as *a*-PAHs, which incorporate extra alkyl groups (Figures S1 and S2). In the K_2_*qn*^1^ layer of the SYY3 well, NAPs include NAP and alkyl NAPs with one-five methyl groups or one ethyl group. The relative contents of alkyl NAPs vary significantly with alkyl carbon number, markedly exceeding that of NAP itself (Table S3). TMNs predominate, with a ratio of TMNs to NAP surpassing 2000. This is followed by DMNs and TeMNs, with MNs and ENs present in lesser quantities. PMNs are the least abundant, with a ratio of PMNs to NAP below 1.0 (Table S3; Figure 8a). The ratios of alkyl NAPs to NAP, including DMNs/NAP, TMNs/NAP, and TeMNs/NAP, exhibit two cycles of increasing magnitude with increasing depth. These ratios are consistently higher in the upper cycle compared to those in the lower cycle (Figure 8a). BIPs consist of BIP and alkyl BIPs with one to two methyl groups. The relative contents are in the order DMBIPs > MBIPs > BIPs (Table S3 and Figure 8b). In most depth intervals of the SYY3 well, the ratios of MBIPs/BIPs and DMBIPs/BIPs are below 6.0, with three zones of high values above 6.0 in the upper, middle, and lower sections, respectively (Figure 8b). PHENs consist of PHEN and alkyl PHENs with one to three methyl groups or one ethyl group. The relative contents in the upper unit are in the order of MPs > PHEN > DMPs + EPs > TMPs, while in the lower unit, they are MPs > DMPs + EPs > PHEN > TMPs (Figure 8c). The ratios of MPs/PHEN, (DMPs + EPs)/PHEN, and TMPs/PHEN exhibit stability in the upper unit, while they show a consistent upward trend with increasing burial depth in the lower unit (Table S3 and Figure 8c). CHRs consist of CHR, MCHRs, and C_2_-alkyl CHRs. The relative contents in the upper unit are mostly in the order of CHR > ≈ MCHRs > C_2_-alkyl CHRs, while in the lower unit, they are MCHRs > CHR > C_2_-alkyl CHRs in its upper subunit and MCHRs > C_2_-alkyl CHRs > CHR in its lower subunit, respectively (Figure 8d). In general, the trends of the ratios MCHRs/CHR and C_2_-alkyl CHRs/CHR with respect to depth are similar to those of MPs/PHEN, (DMPs + EPs)/PHEN, and TMPs/PHEN (Table S3 and Figure 8c,d).

**Figure 9:**
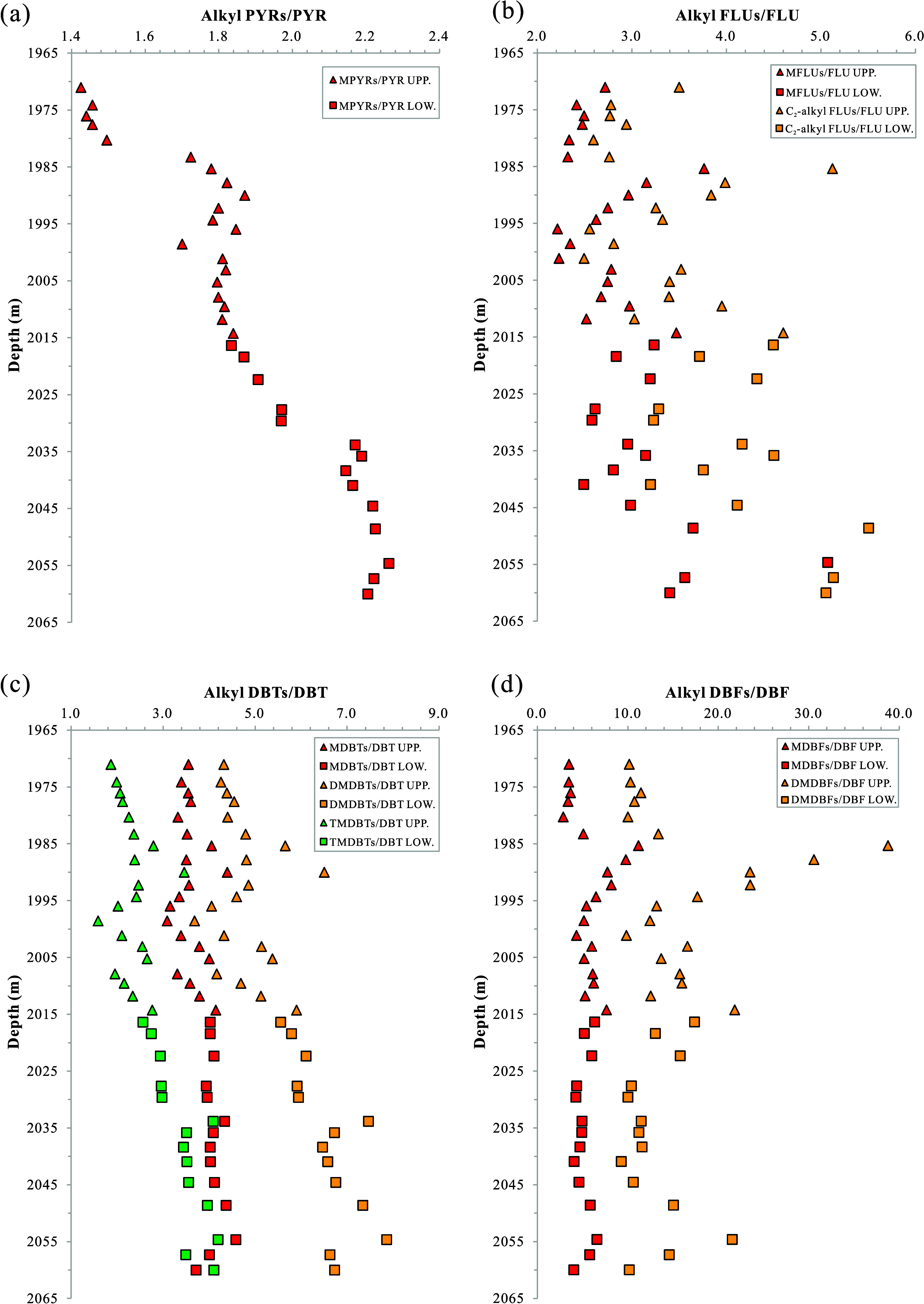
Variation diagrams of content ratios of *a*-PAHs to *p*-PAH of (a) PYRs, (b) FLUs, (c) DBTs, and (d) DBFs with buried depth of the K_2_*qn*^1^ samples from the SYY3 well in the Sanzhao Sag. PYR = pyrene; MPYRs = methylpyrene series; FLU = fluorene; MFLUs = methylfluorene series; C_2_-alkyl FLUs = C_2_ alkyl fluorene series; DBT = dibenzothiophen; MDBTs = methyldibenzothiophen series; DMDBTs = dimethyldibenzothiophene series; TMDBTs = trimethyldibenzothiophene series; DBF = dibenzofuran; MDBFs = methyldibenzofuran series; DMDBFs = dimethyldibenzofuran series.

PYRs in the K_2_*qn*^1^ layer of the SYY3 well are composed of PYR and its methylated derivatives (MPYRs), with MPYRs being more abundant than PYR (Figure 9a). The MPYRs/PYR ratio increases progressively with depth, and this ratio is notably higher in the lower unit compared to the upper unit (Table S4 and Figure 9a). FLUs consist of FLU, methyl FLUs (MFLUs), and C_2_-alkyl FLUs. Their relative abundances are in the order of C_2_-alkyl FLUs > MFLUs > FLU (Figure 9b). Similar to PYRs, the ratios of MFLUs/FLU and C_2_-alkyl FLUs/FLU increase with depth, with higher values observed in the lower unit. However, the sample points observed in the plot are relatively scattered (Figure 9b). DBTs are primarily composed of DBT, methyldibenzothiophenes (MDBTs), dimethyldibenzothiophenes (DMDBTs), and trimethyldibenzothiophenes (TMDBTs). The relative abundances follow the pattern of DMDBTs > MDBTs > TMDBTs > DBTs (Figure 9c). The ratios of MDBTs/DBT, DMDBTs/DBT, and TMDBTs/DBT also increase with depth, with the lower unit showing higher ratios than the upper unit (Table S4 and Figure 9c). DBFs mainly consist of DBF, methyldibenzofurans (MDBFs), and dimethyldibenzofurans (DMDBFs). The relative abundances are in the order of DMDBFs > MDBFs > DBF (Figure 9d). Contrary to other PAHs, the ratios of MDBFs/DBF and DMDBFs/DBF are generally higher in the upper unit than in the lower unit (Table S4 and Figure 9d).

In summary, the composition of PAHs in the SYY3 well exhibits depth-related variations, with the lower unit of the K_2_*qn*^1^ layer showing generally higher ratios of *a*-PAHs to *p*-PAH for most species of PAHs, suggesting an increased degree of alkylation with increasing depth. The alkylation patterns of PHENs, CHRs, PYRs, FLUs, and DBTs show consistent trends, with higher ratios in the lower unit compared to the upper unit (Figures 8c,d and 9a–c). However, NAPs and BIPs, which are bicyclic aromatic hydrocarbons and the lowest molecular weight PAHs, do not follow the same distribution patterns, possibly due to their volatility during extraction processes. Consequently, the observed compositions may not accurately reflect their original distributions. Furthermore, the characteristics of alkylation degree profile evolution in DBFs differ from those in FLUs and DBTs, as evidenced by the generally higher ratio of alkyl DBFs to DBF in the upper unit compared to the lower unit (Figure 9d). The chemical properties of FLUs, DBTs, and DBFs, which have similar molecular structures, are influenced by the carbon, sulfur, and oxygen atoms in their five-membered rings (Figure S3). The alkylation degree of DBTs shows the strongest correlation with depth, followed by FLUs, while DBFs show no clear correlation, possibly suggesting different alkylation mechanisms owing to their different chemical properties. The abundance and distribution of DBFs and their alkyl homologues were regarded to be primarily controlled by the biological sources of hydrocarbon generation.^49^ The MDBFs/DBF ratio, which may reflect paleoclimate changes, indicates a warm climate during certain periods with higher ratios. In the K_2_*qn*^1^ layer of the SYY3 well, the MDBFs/DBF ratio in the upper unit ranges from 2.85 to 11.17 with an average of 5.83, and in the lower unit, it ranges from 4.01 to 6.58 with an average of 5.11 (Table S3). According to the views from the previous study,^49^ the variation of the above ratio in the upper unit suggests a paleoclimate transition from cool to warm and then back to cool. In contrast, the stable ratio in the lower unit (Figure 9d) indicates a more constant paleoclimate. The overall trends of DMDBFs/DBF with depth are similar to those of MDBFs/DBF (Figure 9d), possibly suggesting comparable paleoclimatic implications. Due to the higher percentage content of DMDBFs in DBFs, the fluctuation of the DMDBFs/DBF ratio is more pronounced compared to the MDBFs/DBF ratio (Figure 9d). In conclusion, aside from NAPs, BIPs, and DBFs, the vertical distribution characteristics of the alkylation degree of PAHs generally follow similar patterns.

#### 4.2.2 Main Control of PAH Alkylation

It was thought that the alkylation degree of various PAHs can be influenced by multiple factors, such as the thermal maturity of organic matter, the sedimentary environment, and the biogenetic origin of hydrocarbons.^47,48^ The maturity parameters of poly(methyl)-substituted naphthalenes (*TMNr*, *TeMNr*, and *PMNr*) and phenanthrenes (*MPI1*, *MPI2*, and *MPR*) indicate that the thermal maturity is slightly higher in the lower unit compared to the upper unit (Table S3). In the K_2_*qn*^1^ layer of the SYY3 well, vitrinite reflectance (*R*_o_) values range from 0.82 to 0.89% with an average of 0.86% in the upper unit, and from 0.87% to 0.98% with an average of 0.92% in the lower unit.^65^ The above maturity parameters confirm that the entire K_2_*qn*^1^ layer is in a mature stage with close maturity between the upper and lower units. Therefore, such a minor difference in thermal maturity is insufficient to account for significant disparities in the alkylation of PAHs. It was reported that the content of *a*-PAHs is notably higher than that of the corresponding *p*-PAH in marine crude oils, and the maximum alkylation degrees of NAP, PHEN, FLU, and DBT vary among crude oils of different origins.^48^ Carbonate-sourced crude oils exhibit the highest level of alkylation, typically with C_3_ or C_4_ alkyl-substituted homologues being the most abundant. In contrast, paralic-sourced crude oils show the lowest degree of alkylation, predominantly containing C_1_ or C_2_ alkyl homologues. The alkylation level of PAHs in siliciclastic-sourced crude oils is moderate, with C_2_ or C_3_ alkyl substitutions being the most common. In the K_2_*qn*^1^ samples from the SYY3 well, the most abundant alkyl homologues for NAPs, PHENs, FLUs, and DBTs are C_3_, C_1_, C_2_, and C_2_, respectively, which is overall similar to the alkylation patterns observed in siliciclastic-sourced crude oils. This similarity suggests that the precursor components of kerogen from different source rock types may possess distinct alkylation characteristics in their molecular structures, potentially providing a plausible explanation for the vertical differences in alkylation degrees observed in the K_2_*qn*^1^ samples.

Relative to PAHs, saturated hydrocarbons are more extensively used to indicate geological and geochemical information such as paleoenvironmental conditions and biogenic inputs.^2^ The existing study has delineated the geochemical characteristics of saturated hydrocarbon molecules in the K_2_*qn*^1^ layer of the SYY3 well. Significant differences were revealed in key geochemical parameters of saturated hydrocarbons, which are indicative of depositional environments and biotic inputs, between the upper and lower units of the K_2_*qn*^1^ layer.^65^ The typical depositional environmental parameters include Pr/Ph, GR/C_30_H, C_29_ 18α(H),21β(H)-30-norneohopane to C_29_ nohopane (C_29_Ts/C_29_NH), C_27_ 22,29,30-trisnorneohopane to C_27_ 22,29,30-trisnorhopane (Ts/Tm), and pregnane and homopregnane to regular steranes ((PR+HPR)/RST). The Pr/Ph ratio provides insight into the redox conditions present within the sedimentary environment, while the GR/C_30_H ratio is indicative of salinity or stratification levels, typically with higher values implying higher salinity.^2,86^ Under conditions of similar maturity, the ratios C_29_Ts/C_29_NH and Ts/Tm are particularly responsive to clay minerals, with elevated values in environments rich in clay minerals.^2,65^ The Ts/Tm ratios in our samples for the upper and lower units are 1.21–2.76 (avg. 1.98) and 2.12–4.83 (avg. 4.16), respectively.^65^ Such a marked disparity cannot be solely due to minor thermal maturity variations. Hence, it is crucial to note that Ts/Tm can indicate clay content in sedimentary environments rather than maturity in these samples. The (PR+HPR)/RST ratio was regarded to be correlated with the lithology of source rocks and the sedimentary environment’s clay mineral content.^87^ The representative biotic input parameters comprise the ratios of C_19–20_ tricyclic terpane to C_21–26_ tricyclic terpane (C_19–20_TT/C_21–26_TT), C_24_ tetracyclic terpane to C_26_ tricyclic terpane (C_24_TE/C_26_TT), regular steranes to 17α(H)-hopane (RST/αH), C_27_ 5α(H), 14α(H), 17α(H) sterane 20R to C_29_ 5α(H), 14α(H), 17α(H) sterane 20R (C_27_R/C_29_R), and C_30_ 4-methylsteranes to C_27_-29 5α(H), 14α(H), 17α(H) steranes 20R (4-MST/20RαααRST).^65^ The ratios C_19–20_TT/C_21–26_TT and C_24_TE/C_26_TT are indicative of the biotic contributions from terrestrial higher plants to source rocks.^88,89^ The RST/αH ratio reflects the relative contributions of eukaryotic organisms (primarily algae and higher plants) versus prokaryotic organisms (predominantly bacteria) to the source rocks.^2^ Generally, the C_27_R/C_29_R ratio was usually used to reflect the relative biotic input contributions of eukaryotic algae and terrestrial higher plants.^2^ However, in the case of the K_2_*qn*^1^ samples, the C_29_ steranes were believed to mainly originate from prokaryotic organisms.^65^ Therefore, here, the C_27_R/C_29_R ratio is used to indicate the relative contributions of eukaryotic algae and prokaryotic organisms. The 4-MST/20RαααRST ratio demonstrates a strong negative correlation with the C_27_R/C_29_R ratio, indicating that 4-methylsteranes in our samples likely stem from prokaryotic organisms rather than the eukaryotic algae typically assumed.^65^ Consequently, the 4-MST/20RαααRST ratio is indicative of input from prokaryotic biological sources.

**Figure 10:**
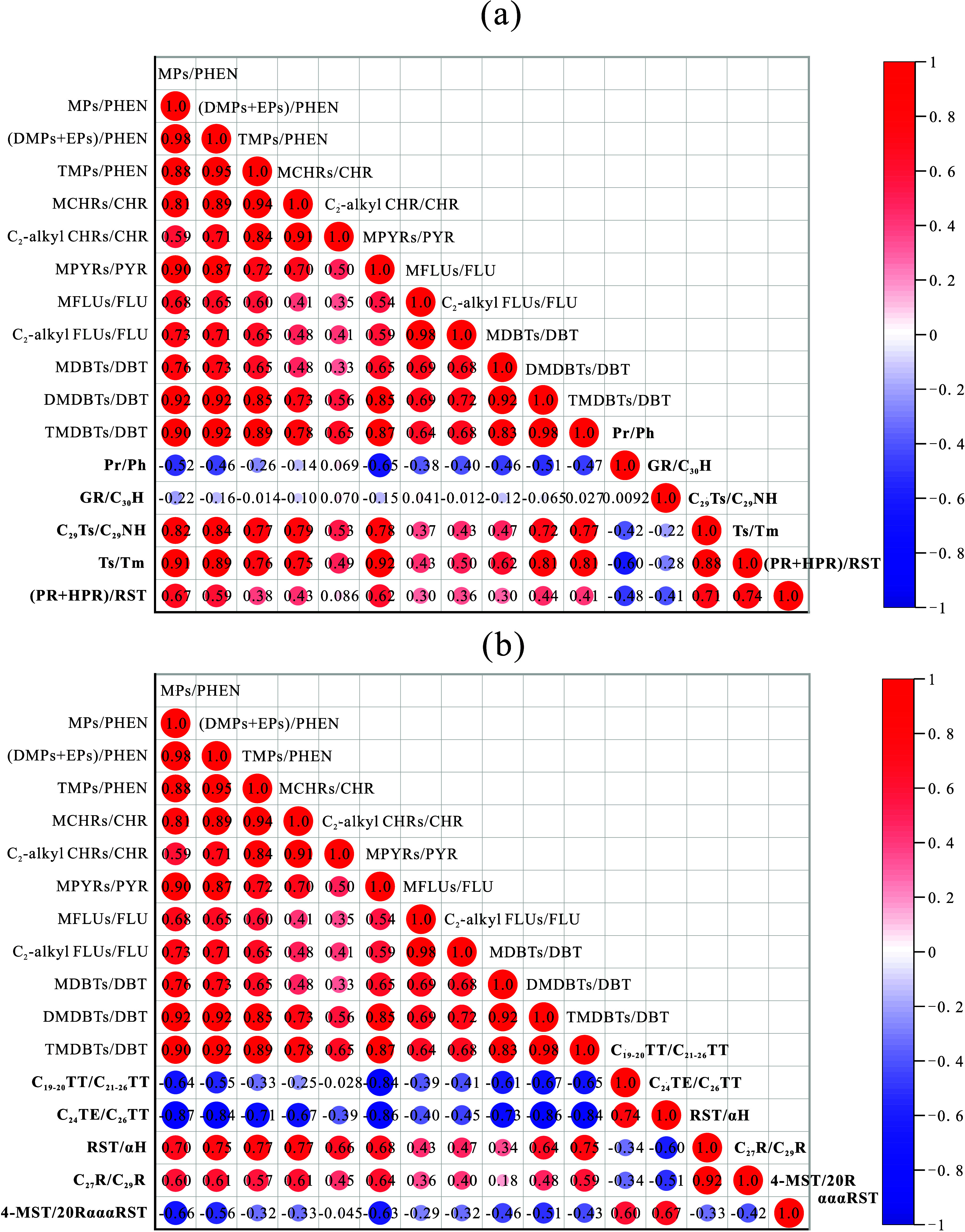
Correlation matrixes of the content ratios of *a*-PAHs to *p*-PAH and typical saturated geochemical indexes about (a) the sedimentary environment and (b) the biotic input of the K_2_*qn*^1^ samples from the SYY3 well in the Sanzhao Sag. Data of the indexes marked in bold are saturated geochemical indexes referred from the published paper^65^ with the exact meaning as follows. Reprinted (Adapted or Reprinted in part) with permission from [Xiao, F.; Yang, J.; Li, S.; Yao, Y.; Huang, Y.; Gao, X. Enrichment and movability of lacustrine tight shale oil for the first member of the Upper Cretaceous Qingshankou Formation in the Sanzhao Sag, Songliao Basin, NE China: Insights from saturated hydrocarbon molecules. Fuel 2024, 368, 131615. 10.1016/j.fuel.2024.1316 15.] Copyright [2024] [Elsevier]. Pr/Ph = pristane/phytane; GR/C_30_H = gammacerane/C_30_ hopane; C_29_ Ts/C_29_ NH = C_29_ 18α(H),21β(H)-30- norneohopane/C_29_ nohopne; Ts/Tm = C_27_ 22,29,30-trisnorneohopane/C_27_ 22,29,30-trisnorhopane; (PR+HPR)/RST = (pregnane + homopregnane)/regular steranes; TT = tricyclic terpane; TE = tetracyclic terpane; RST = regular steranes; αH = 17α(H)-hopane; C_27_R/C_29_R = C_27_ 5α(H), 14α(H), 17α(H) sterane (20R)/C_29_ 5α(H), 14α(H), 17α(H) sterane (20R); 4-MST/20RαααST = C_30_ 4-methylsteranes/C_27_-29 5α(H), 14α(H), 17α(H) steranes (20R).

Correlation analyses were performed to assess the relationship between the above molecular geochemical parameters of saturated hydrocarbons and PAH alkylation parameters, including alkyl PHENs/PHEN, alkyl CHRs/CHR, alkyl PYRs/PYR, alkyl FLUs/FLU, and alkyl DBTs/DBT (Figure 10). Upon comparison with the sedimentary environmental parameters associated with saturated hydrocarbons (Figure 10a), our findings reveal that the PAH alkylation parameters show a strong positive correlation with the C_29_Ts/C_29_NH and Ts/Tm ratios, a moderate positive correlation with the (PR+HPR)/RST ratio, and a weak negative correlation with the Pr/Ph ratio. Notably, there is no significant correlation observed with the GR/C_30_H ratio. These results suggest that the catalysis by clay minerals predominantly governs the alkylation degree of PAHs and that a reducing environment is also a favorable factor, despite the alkylation degree of PAHs being independent of water salinity. Yang et al.^60^ reported that in the SYY3 well, the lower unit of the K_2_*qn*^1^ layer has a clay mineral content ranging from 7.30 to 41.00%, with an average of 28.46%; the upper unit has a clay mineral content ranging from 7.10 to 38.80%, with an average of 17.90%. The significantly higher clay mineral content in the lower unit confirms that clay minerals can be decisive factors in the alkylation of PAHs. The analysis presented above indicates that the differences in redox potential and salinity between the upper and lower units of the K_2_*qn*^1^ layer in the SYY3 well are not markedly distinct. This observation is corroborated by the overlapping distribution of sample points (Figure 7a–c), which also suggests that neither the redox potential of the sedimentary environment nor the salinity of water is the primary determinant for the alkylation of PAHs. In comparison with biotic input parameters of saturated hydrocarbons, PAH alkylation parameters are positively correlated with RST/αH and C_27_R/C_29_R, and negatively correlated with C_19–20_TT/C_21–26_TT, C_24_TE/C_26_TT, and 4-MST/20RαααRST (Figure 10b). The findings indicate that a higher biogenic input from eukaryotic algae and a lower input from terrestrial higher plants and prokaryotes favor the alkylation of PAHs. Collectively, the enhanced alkylation of PAHs observed in the lower unit of the K_2_*qn*^1^ layer is primarily attributed to the clay-rich sedimentary conditions coupled with a greater presence of eukaryotic algae and a reduced presence of terrestrial higher plants and prokaryotes, which contribute to the elevated formation of alkyl PAHs.

### 4.3 Differential Distribution of Three Pairs of p-PAH Isomers

#### 4.3.1 Profile Variation of *p*-PAH Isomerization

**Figure 11:**
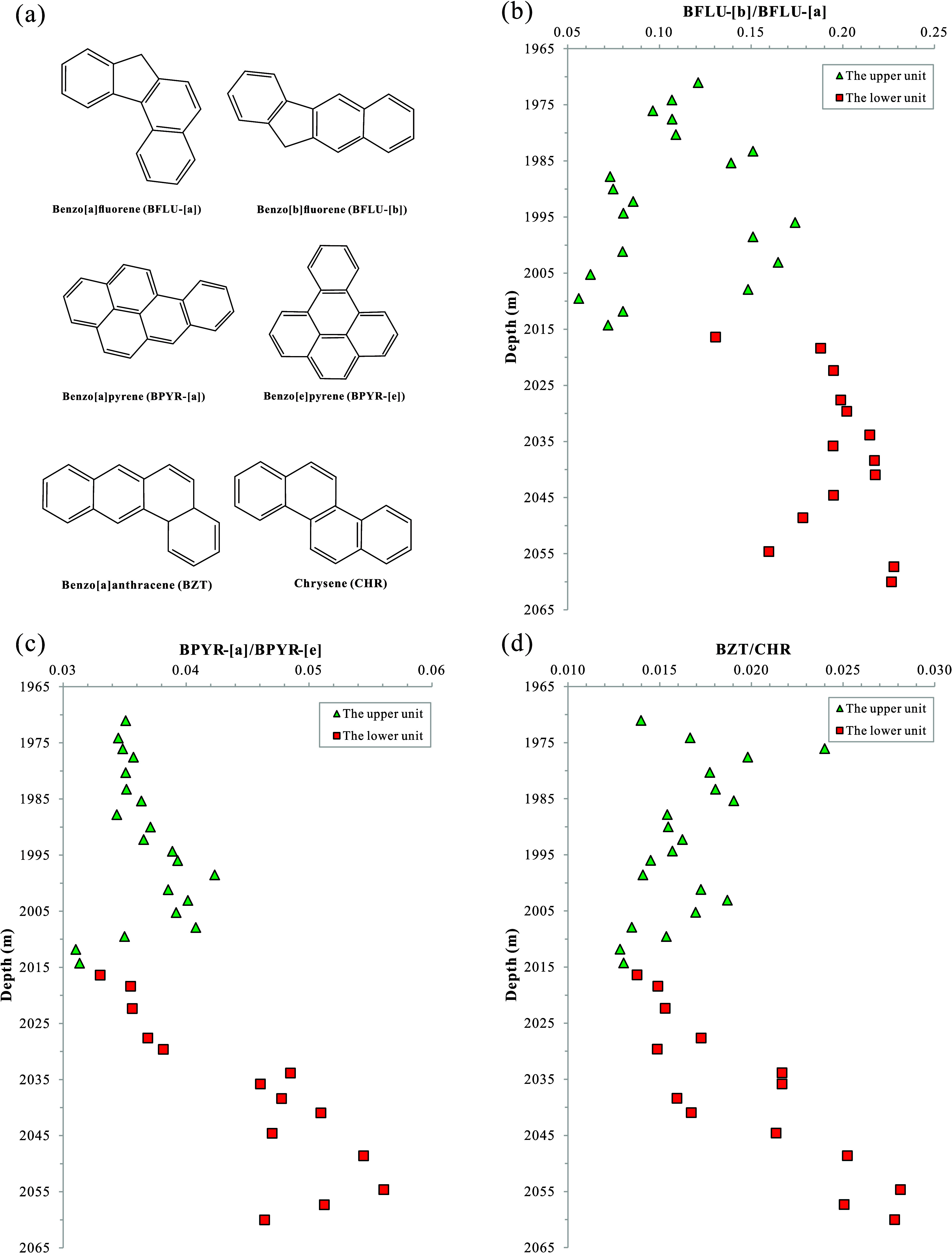
(a) Schematic diagrams of molecular structures of BFLUs, BPYRs, BZT, and CHR variation diagrams of content ratios of parent PAH isomers including (b) BFLU-[b]/BFLU-[a], (c) BPYR-[a]/BPYR-[e], and (d) BZT/CHR with buried depth of the K_2_*qn*^1^ samples from the SYY3 well in the Sanzhao Sag. BFLU-[a] = benzo[a]fluorene; BFLU-[b] = benzo[b]fluorene; BPYR-[a] = benzo[a]pyrene; BPYR-[e] = benzo[e]pyrene; BZT = benzo[a]anthracene; CHR = chrysene.

In the soluble organic matter of the K_2_*qn*^1^ layer from the SYY3 well, *p*-PAHs exhibit multiple pairs of isomers, including benzo[a]fluorene (BFLU-[a]) and benzo[b]fluorene (BFLU-[b]), benzo[a]pyrene (BPYR-[a]) and benzo[e]pyrene (BPYR-[e]), as well as BZT and CHR (Figure 11a). Our samples reveal a significantly lower abundance of BFLU-[b] compared to BFLU-[a], with the BFLU-[b]/BFLU-[a] ratio consistently below 0.25 (Table S4). Benzo[a]carbazole and benzo[b]carbazole have molecular structures similar to those of BFLU-[a] and BFLU-[b]. Due to its linear substitution pattern, benzo[b]carbazole possesses greater internal energy and reduced thermal stability compared to benzo[a]carbazole, as demonstrated by the previous studies.^90,91^ By extension, it is postulated that BFLU-[b] exhibits lower thermal stability than BFLU-[a]. This inferred disparity in thermal stability can account for the observed low BFLU-[b]/BFLU-[a] ratio within the samples. Within the K_2_*qn*^1^ layer, the BFLU-[b]/BFLU-[a] ratio ranges from 0.06 to 0.17 with an average of 0.11 in the upper unit, and from 0.13 to 0.23 with an average of 0.20 in the lower unit (Table S4 and Figure 11b). Despite the slightly higher thermal maturity in the lower unit, the unexpectedly higher BFLU-[b] content suggests that thermal maturity is not the factor influencing the composition and distribution of BFLUs.

BPYR-[e] has strong resistance to weathering, migration, and diagenetic alteration, while BPYR-[a] and BZT are less stable in geological processes.^92^ Accordingly, the abundances of BPYR-[a] and BZT are notably low, with the BPYR-[a]/BPYR-[e] and BZT/CHR ratios below 0.06 and 0.03, respectively (Table S4). In the K_2_*qn*^1^ layer, the BPYR-[a]/BPYR-[e] ratio varies from 0.031 to 0.042 with an average of 0.037 in the upper unit, and from 0.033 to 0.056 with an average of 0.045 in the lower unit (Table S4 and Figure 11c). Similarly, the BZT/CHR ratio ranges from 0.013 to 0.024 with an average of 0.016 in the upper unit and from 0.014 to 0.028 with an average of 0.020 in the lower unit (Table S4 and Figure 11d). The elevated ratios of BPYR-[a]/BPYR-[e] and BZT/CHR in the lower unit, similar to those observed in BFLU-[b]/BFLU-[a], suggest a higher content of less stable p-PAH isomers within this sublayer.

#### 4.3.2 Main Control of *p*-PAH Isomerization

**Figure 12:**
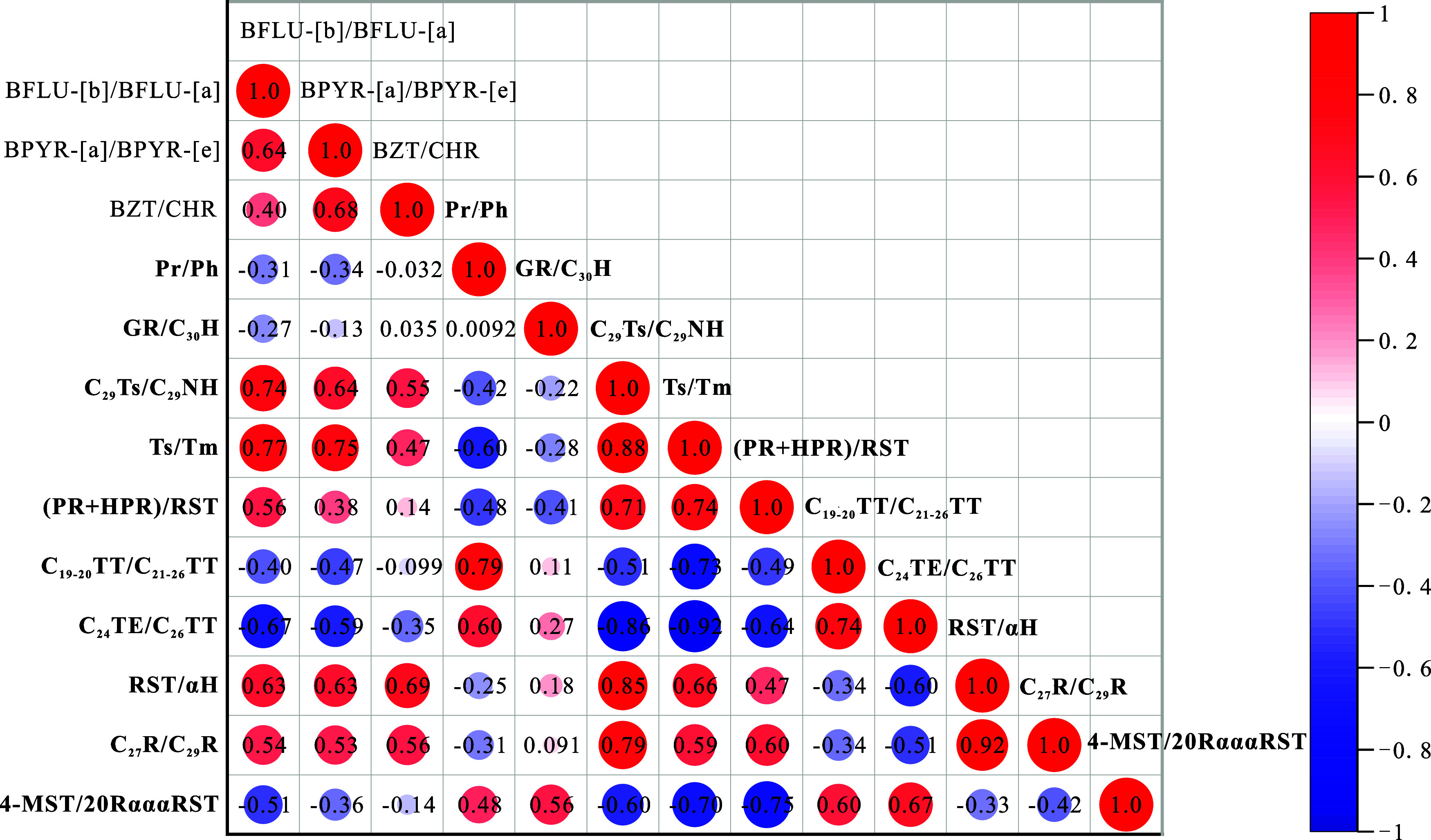
Correlation matrixes of content ratios of *p*-PAH isomers including BFLU-[b]/BFLU-[a], BPYR-[a]/BPYR-[e], and BZT/CHR and the typical saturated geochemical indexes about sedimentary environment and biotic input of the K_2_*qn*^1^ samples from the SYY3 well in the Sanzhao Sag. Data of the indexes marked in bold are saturated geochemical indexes referred from the published paper^65^ with the exact meaning listed in the caption of Figure 10. Reprinted (Adapted or Reprinted in part) with permission from [Xiao, F.; Yang, J.; Li, S.; Yao, Y.; Huang, Y.; Gao, X. Enrichment and movability of lacustrine tight shale oil for the first member of the Upper Cretaceous Qingshankou Formation in the Sanzhao Sag, Songliao Basin, NE China: Insights from saturated hydrocarbon molecules. Fuel 2024, 368, 131615. 10.1016/j.fuel.2024.1316 15.] Copyright [2024] [Elsevier].

Correlation analysis between the PAH isomer ratios (BFLU-[b]/BFLU-[a], BPYR-[a]/BPYR-[e], and BZT/CHR) and the geochemical parameters of saturated hydrocarbon molecules was conducted. These PAH isomer ratios exhibit weak relationships with Pr/Ph and GR/C_30_H but show positive correlations with C_29_Ts/C_29_NH, Ts/Tm, (PR+HPR)/RST, RST/αH, and C_27_R/C_29_R, and negative correlations with C_19–20_TT/C_21–26_TT, C_24_TE/C_26_TT, and 4-MST/20RαααRST (Figure 12). This suggests that the ratios of PAH isomers are predominantly regulated by the presence of clay minerals and biological input with minimal influence from the redox properties and salinity of the sedimentary environment. In essence, the formation and preservation of BFLU-[b], BPYR-[a], and BZT within *p*-PAHs are likely favored in clay-rich sedimentary environments characterized by a higher proportion of eukaryotic algae and a lower proportion of terrigenous higher plants and prokaryotes. Overall, the factors influencing the isomerization of *p*-PAHs and alkylation of PAHs in the shale strata are similar.

## 5 Conclusions

1. The comprehensive analysis of thirty-four core samples with close thermal maturity retrieved from the SYY3 well within the K_2_*qn*^1^ layer of the Sanzhao Sag has revealed distinct differences in the composition and distribution of PAHs between the upper and lower units. In comparison to the lower unit, the upper unit of the K_2_*qn*^1^ shale strata in the SYY3 well generally exhibits lower PAH concentrations, along with a significant decrease in DBTs. This trend suggests that the sedimentary environment was more oxidative and less conducive to biological productivity. The analysis of PAHs indicates that, in the SYY3 well, the upper unit of the K_2_*qn*^1^ shale strata has a higher proportion of biogenic contributions from terrestrial higher plants and prokaryotes than the lower unit. Conversely, it has a lower proportion of contributions from lower aquatic organisms.
2. The lower unit exhibits enhanced alkylation degrees of PHENs, CHRs, PYRs, FLUs, and DBTs, and higher *p*-PAH isomer ratios, including BFLU-[b]/BFLU-[a], BPYR-[a]/BPYR-[e], and BZT/CHR. Correlation analyses between the alkylation degrees of PAHs and the ratios of *p*-PAH isomers with geochemical parameters of saturated hydrocarbons underscore the pivotal role of the catalysis by clay minerals and biotic input in dictating the PAH alkylation and isomer distribution of *p*-PAHs. Our findings revealed that the salinity exerts no obvious influence on that. While the reductive conditions of the sedimentary environment appear to slightly enhance the alkylation of these PAHs, they do not exert a notable effect on their isomerization of *p*-PAHs.
3. The alkylation degrees of PAHs and the ratios of *p*-PAH isomers can serve as robust indicators of the variation of mineralogy and biotic contributions to hydrocarbon generation. Nevertheless, there is a need for future research to delve into the mechanisms behind the alkylation of PAHs and the isomerization of their parent compounds at comparable stages of maturity.
4. The above insights provide systematic PAH molecular geochemical evidence for studying the heterogeneity of the K_2_*qn*^1^ source rock in terms of depositional environment and the source of hydrocarbon-generating kerogen, the analysis of crude oil sources under similar thermal maturity, and the selection of sweet spots in shale oil strata in the Songliao Basin.

## Supporting Information Available

Mass chromatograms of the PAHs including NAPs, PHENs, BZT, CHRs, BPYRs, BFRT, FRT, PYRs, and BFLUs (Figure S1), and FLUs, DBTs, BIPs, DBFs, and TASs (Figure S2); basic molecular structure diagrams of major PAHs (Figure S3); relative percentage contents of the major PAHs excluding NAPs and BIPs (Table S1); common aromatic geochemical indexes and typical saturated geochemical indexes indicating biotic input, sedimentary environment, and thermal maturity (Table S2); content ratios of *a*-PAHs to *p*-PAH of NAPs, BIPs, PHENs, and CHRs (Table S3); content ratios of *a*-PAHs to *p*-PAH of PYRs, FLUs, DBTs, and DBFs; and three main ratios of *p*-PAH isomers (Table S4) (PDF)

## References

[ref1] de Hemptinne J.-C.; Peumery R.; Ruffier-Meray V.; Moracchini G.; Naiglin J.; Carpentier B.; Oudin J. L.; Connan J. Compositional changes resulting from the water-washing of a petroleum fluid. J. Pet. Sci. Eng. 2001, 29, 39–51. 10.1016/S0920-4105(00)00089-9.

[ref2] Peters K. E.; Walters C. C.; Moldowan J. M. The Biomarker Guide: Vol. 2, Biomarkers and Isotopes in Petroleum Systems and Earth History, 2nd ed.; Cambridge University Press: Cambridge, UK, 2005.

[ref3] Li Y.; Jiang Q.; Liu X.; Wang J.; Han M.; Li X. Identification methods and the main factors controlling water washing in secondary oil reservoirs: A case study of Qingshuihe formation of lower Cretaceous in Mosuowan area of Junggar Basin. Geoenergy Sci. Eng. 2023, 224, 211538 10.1016/j.geoen.2023.211538.

[ref4] Li Z.; Feng Z.; Song G.; Wang X. Distribution and composition of aromatic hydrocarbons and classification of oil in Songliao basin. Oil Gas Geol. 2005, 26 (4), 494–500.

[ref5] Meng J.; Liu L.; Zhang M.; Wang Y. Indicative function of aromatic hydrocarbon in crude oil on depositional environment. J. China Univ. Min. Technol. 2011, 40 (6), 901–907.

[ref6] Xiang T.; Ma F.; Pan K. Effect of mild-to-moderate biodegradation on alkyl naphthalene and alkyl phenanthrene in crude oil. J. Xi’an Shiyou Univ. 2012, 27 (1), 81–86.

[ref7] Zhu Y. Geochemical characteristics of aromatic hydrocarbon from oil in Tarim Basin. Geochimica 1998, 25 (1), 10–18.

[ref8] Zhu Y.; Zhang H.; Fu J.; Sheng G. Distribution and composition of aromatic hydrocarbon in various oils from Tarim Basin. Acta Petrolei Sin. 1998, 19 (3), 33–37.

[ref9] Guo X.; He S.; He W. Aromatic geochemistry characteristics of light oils from Panyu Lower Uplift in Pearl River Mouth Basin. Acta Petrolei Sin. 2008, 29 (1), 52–57.

[ref10] Jia C.; Wang Y.; Gu Y.; Huang J. Geochemical characteristics of aromatic hydrocarbons of crude oils from Ordovician reservoir in the Tahe Oilfield. Pet. Geol. Exp. 2009, 31 (4), 384–388. 10.11781/sysydz200904384.

[ref11] Chen X.; Zhang M.; Cheng X. Geochemical characteristics of aromatic hydrocarbons of crude oil in Qinjiatun Oilfield of Lishu Fault Depression in the Southern Songliao Basin. J. Oil Gas Technol. 2013, 35 (8), 28–48.

[ref12] Liu Y.; Gang W.; Chen G.; Chen J.; Jiang C. Geochemical characteristics of aromatic hydrocarbons of Chang7 source rocks from the Yanchi-Dingbian area, Ordos Basin. Acta Sedimentol. Sin. 2018, 36 (4), 818–828.

[ref13] Tang Y.; Zheng L.; Li Y.; Gao X.; Zong W.; Sun Q.; He D. The aromatic fraction of source rocks from the Jurassic Haifanggou Formation in Niuyingzi Depression of Lingyuan-Ningcheng Basin. Nat. Gas Geosci. 2019, 30 (3), 433–446.

[ref14] Meng B.; Zhou S.; Li J.; Chen K.; Chen C.; Li P.; Sun Z. Distribution characteristics and significance of the aromatic hydrocarbons molecular biomarker in crude oil from the northwestern Qaidam Basin. Nat. Gas Geosci. 2021, 32 (5), 738–753.

[ref15] Cao J.; Wan Y.; Zhang B.; Song C.; Sun W. Study on characteristics of aromatic compounds in source rocks of Lower Permian Zhanjin Formation in the Central Uplift of Qiangtang Basin. Coal Geol. China 2023, 35 (11), 23–30.

[ref16] Akinlua A.; Dada O. P.; Usman F. O.; Adekola S. A. Source rock geochemistry of central and northwestern Niger Delta: Inference from aromatic hydrocarbons content. Energy Geosci. 2023, 4, 100141 10.1016/j.engeos.2022.100141.

[ref17] Xu H.; George S. C.; Hou D. Algal-derived polycyclic aromatic hydrocarbons in Paleogene lacustrine sediments from the Dongying Depression, Bohai Bay Basin. China. Mar. Pet. Geol. 2019, 102, 402–425. 10.1016/j.marpetgeo.2019.01.004.

[ref18] Xie W.; Jia J.; Liu Z. Distribution and geological significance of polycyclic aromatic hydrocarbons in the coal from the Shimengou Formation of Middle Jurassic in the northern Qaidam basin. Acta Geol. Sin. 2023, 97 (7), 2363–2377. 10.19762/j.cnki.dizhixuebao.2022049.

[ref19] Hossain H. M. Z.; Sampei Y.; Roser B. P. Polycyclic aromatic hydrocarbons (PAHs) in late Eocene to early Pleistocene mudstones of the Sylhet succession, NE Bengalbasin, Bangladesh: Implications for source and paleoclimate conditions during Himalayan uplift. Org. Geochem. 2013, 56, 25–39. 10.1016/j.orggeochem.2012.12.001.

[ref20] Zakrzewski A.; Kosakowski P.; Waliczek M.; Kowalski A. Polycyclic aromatic hydrocarbons in Middle Jurassic sediments of the Polish basin provide evidence for high-temperature palao-wildfires. Org. Geochem. 2020, 145, 104037 10.1016/j.orggeochem.2020.104037.

[ref21] Strachan M. G.; Alexander R.; Kagi R. I. Trimethylnaphthalenes in crude oils and sediments: effects of source and maturity. Geochim. Cosmochim. Acta 1988, 52 (5), 1255–1264. 10.1016/0016-7037(88)90279-7.

[ref22] van Aarssen B. G. K.; Bastow T. P.; Alexander R.; Kagi R. I. Distributions of methylated naphthalenes in crude oils: indicators of maturity, biodegradation and mixing. Org. Geochem. 1999, 3 (10), 1213–1227. 10.1016/S0146-6380(99)00097-2.

[ref23] Yang Y.; Zhang Z.; Fang C.; Hu Y.; Li W.; Qin L. Characteristics of triaromatic steroids and oil-source correlation of the crude oils in the Qintong Sag, Subei Basin. In The 4th International Symposium on Hydrocarbon Accumulation Mechanism and Resource Evaluation, Beijing, 2006.

[ref24] Hughes W. B. Use of thiophenic organosulfur compounds in characterizing crude oils derived from carbonate versus siliciclastic sources. In Petroleum Geochemistry and Source Rock Potential of Carbonate Rocks; AAPG Studies in Geology, 1984; Vol. 18, pp 181–196.

[ref25] Moldowan J. M.; Seifert W. K.; Gallegos E. J. Relationship between petroleum composition and depositional environment of petroleum source rocks. AAPG Bull. 1985, 69, 1255–1268. 10.1306/AD462BC8-16F7-11D7-8645000102C1865D.

[ref26] Li S.; He S. Geochemical characteristics of dibenzothiophene, dibenzofuran and fluorine and their homologues and their environmental indication. Geochimica 2008, 37 (1), 45–50.

[ref27] Li M.; Shi S.; Wang T.-G.; Zhang L.; Yang F. Several problems on the use of relative abundances of fluorenes, dibenzofurans and dibenzothiophenes in the study of depositional environment. Adv. Geosci. 2013, 3, 22–28. 10.12677/ag.2013.31004.

[ref28] Hughes W. B.; Holba A. G.; Dzou L. I. P. The ratio of dibenzothiophene to phenanthrene and pristane to phytane as indicators of depositional environment and lithology of petroleum source rocks. Geochinica et Cosmochimica Acta 1995, 59 (17), 3581–3598. 10.1016/0016-7037(95)00225-O.

[ref29] Alexander R.; Kagi R. I.; Sheppard P. N. 1,8-Dimethylnaphthalene as an indicator of petroleum maturity. Nature 1984, 308, 442–443. 10.1038/308442a0.

[ref30] Alexander R.; Kagi R. I.; Rowland S. J.; Sheppard P. N.; Chirila T. V. The effects of thermal maturity on distribution of dimethylnaphthalenes and trimethylnaphthalenes in some ancient sediments and petroleums. Geochim. Cosmochim. Acta 1985, 49, 385–395. 10.1016/0016-7037(85)90031-6.

[ref31] Radke M.; Welte D. H.; Willsch H. Geochemical study on a well in the Western Canada Basin: relation of aromatic distribution pattern to maturity of organic matter. Geochim. Cosmochim. Acta 1982, 46, 1–10. 10.1016/0016-7037(82)90285-X.

[ref32] Radke M.; Willsch H.; Welte D. H. Maturity parameters based on aromatic hydrocarbons: influence of the organic matter type. Org. Geochem. 1986, 10, 51–63. 10.1016/0146-6380(86)90008-2.

[ref33] Kvalheim O.; Christy A. A.; Telnaes N.; Bjo̷rseth A. Maturity determination of organic matter in coals using the methylphenanthrene distribution. Geochim. Cosmochim. Acta 1987, 51, 1883–1888. 10.1016/0016-7037(87)90179-7.

[ref34] Bao J.; Wang T.-G.; Zhou Y.; Yu F.; Wang J.; Chen F. J. The relationship between methyl phenanthrene ratios and the evolution of organic matter. J. Jianghan Pet. Inst. 1992, 14 (4), 8–13.

[ref35] Chakhmakhchev A.; Suzuki M.; Takayama K. Distribution of alkylated dibenzothiophenes in petroleum as a tool for maturity assessments. Org. Geochem. 1997, 26 (7), 483–490. 10.1016/S0146-6380(97)00022-3.

[ref36] Santamaria-Orozco D.; Horsfield B.; di Primio R.; Welte D. H. Influence of maturity on distributions of benzo- and dibenzothiophenes in Tithonian source rocks and crude oils, Sanda de Campeche. Mexico. Org. Geochem. 1998, 28 (7–8), 423–439. 10.1016/S0146-6380(98)00009-6.

[ref37] Xiao F.; Liu L.; Zhang Z.; Wu K.; Xu Z. J.; Zhou C. Conflicting sterane and aromatic maturity parameters in Neogene light oils, eastern Chepaizi High, Junggar Basin. NW China. Org. Geochem. 2014, 76, 48–61. 10.1016/j.orggeochem.2014.07.014.

[ref38] Luo Q.; George S.; Xu Y.; Zhong N. Organic geochemical characteristics of the Mesoproterozoic Hongshuizhuang Formation from northern China: implications for thermal maturity and biological sources. Org. Geochem. 2016, 99, 23–37. 10.1016/j.orggeochem.2016.05.004.

[ref39] Ni C.; Bao J.; Gu Y. Study of biodegradation effect on aromatic biomarker parameters. Pet. Geol. Exp. 2008, 30 (4), 386–389. 10.11781/sysydz200804386.

[ref40] Zhan Z.; Bao J.; Zhu C.; Yuan L.; Xu W.; Shi Y. Influence of biodegradation on the compositional characteristics of fluorene, dibenzofuran and dibenzothiophene and their methyl homologues in crude oils from the Liaohe Basin. Acta Sedimentol. Sin. 2010, 28 (1), 194–200.

[ref41] Wang T.-G.; He F.; Li M.; He Y.; Guo S. Alkyl dibenzothiophenes: Molecular markers for tracing reservoir charging pathways. Chin. Sci. Bull. 2005, 50 (2), 176–182.

[ref42] Zhang L.; Li M.; Yang F. Progress of geochemical research on dibenzofuran and its chemical mechanism as molecular tracer for oil charging pathways. Oil Gas Geol. 2012, 33 (4), 633–639.

[ref43] Radke M. Application of aromatic compounds as maturity indicators in source rocks and crude oils. Mar. Pet. Geol. 1988, 5, 224–236. 10.1016/0264-8172(88)90003-7.

[ref44] Alexander R.; Bastow T. P.; Fisher S. J.; Kagi R. I. Geosynthesis of organic compounds: II. Methylation of phenanthrene and alkylphenanthrenes. Geochim. Cosmochim. Acta 1995, 59, 4259–4266. 10.1016/0016-7037(95)00285-8.

[ref45] Zheng T.; Zhang Q.; Deng E. Applications of low molecular weight polycyclic aromatic hydrocarbons (PAHs) and diamondoids ratios/indices on the maturity assessment of coal-bearing source rock: Insight from mature to overmature Longtan Shale. Int. J. Coal Geol. 2023, 269, 104209 10.1016/j.coal.2023.104209.

[ref46] Zheng X.; Schwark L.; Stockhausen M.; Luo Q.; Wu J.; Zhong N.; Schovsbo N. H.; Sanei H. Effects of synthetic maturation on phenanthrenes and dibenzothiophenes over a maturity range of 0.6 to 4.7% EASY%Ro. Mar. Pet. Geol. 2023, 153, 106285 10.1016/j.marpetgeo.2023.106285.

[ref47] Zhou S.; Jia X.; Song Z.; Wang B.; Zou H.; Shi J. Aromatic compositions of different occurrence states in deep carbonate rocks from Tarim Basin. Acta Sedimentol. Sin. 2008, 26 (2), 330–339.

[ref48] Requejo A. G.; Sassen R.; McDonald T.; Denoux G.; Kennicutt M. C. II; Brooks J. M. Polynuclear aromatic hydrocarbons (PAH) as indicators of the source and maturity of marine crude oils. Org. Geochem. 1996, 24 (10/11), 1017–1033. 10.1016/S0146-6380(96)00079-4.

[ref49] Jiang L.; Nytoft H. P.; Kampoli I.; George S. C. Distribution, occurrence and identification of dibenzofuran, benzo[b]naphthofurans and their alkyl derivatives in Gippsland Basin source rocks. Org. Geochem. 2024, 187, 104708 10.1016/j.orggeochem.2023.104708.

[ref50] Cassani F.; Gallango O.; Talukdar S.; Vallejos C.; Ehrmann U. Methylphenanthrene maturity index of marine source rock extracts and crude oils from the Maracaibo Basin. Org. Geochem. 1988, 13, 73–89. 10.1016/0146-6380(88)90027-7.

[ref51] Requejo A. G. Maturation of petroleum source rocks—II. Quantitative changes in extractable hydrocarbon content and composition associated with hydrocarbon generation. Org. Geochem. 1994, 21 (1), 91–105. 10.1016/0146-6380(94)90089-2.

[ref52] Szczerba M.; Rospondek M. J. Controls on distributions of methylphenanthrenes in sedimentary rock extracts: critical evaluation of existing geochemical data from molecular modelling. Org. Geochem. 2010, 41 (12), 1297–1311. 10.1016/j.orggeochem.2010.09.009.

[ref53] Sun L.; Liu H.; He W.; Li G.; Zhang S.; Zhu R.; Jin X.; Meng S.; Jiang H. An analysis of major scientific problems and research paths of Gulong shale oil in Daqing Oilfield, NE China. Pet. Explor. Dev. 2021, 48 (3), 527–540. 10.1016/S1876-3804(21)60043-5.

[ref54] Xiao F.; Yang J.; Li S.; Yao Y.; Li A.; Zhang L.; Huang Y. Oil-bearing parameter optimization and resource calculation of the shale oil in Qijia and Gulong Sags, Songliao Basin. Geol. Resour. 2021, 30 (3), 395–404. 10.13686/j.cnki.dzyzy.2021.03.022.

[ref55] Yang J.; Li S.; Yao Y.; Xiao F.; Li A.; Zhang L.; Huang Y. Strategic survey results of shale oil in the first member of Qingshankou Formation, Upper Cretaceous in northern Songliao Basin. Geol. Resour. 2021, 30 (3), 232–238. 10.13686/j.cnki.dzyzy.2021.03.004.

[ref56] Yao Y.; Xiao F.; Li S.; Yang J.; Huang Y.; Ye C. Enrichment mode of shale oil in the first Member of Cretaceous Formation in the southern Qijia sag, Songliao Basin. Acta Geol. Sin. 2024, 10.19762/j.cnki.dizhixuebao.2024423.

[ref57] Bai J.; Xu X.; Liu W.; Zhao W.; Jiang H. Paleoenvironmental Evolution and Organic Matter Enrichment Genesis of the Late Turonian Black Shale in the Southern Songliao Basin. NE China. Acta Geol. Sin. 2024, 98 (5), 1338–1358. 10.1111/1755-6724.15209.

[ref58] Liu B.; Shi J.; Fu X.; Lyu Y.; Sun X.; Gong L.; Bai Y. Petrological characteristics and shale oil enrichment of lacustrine fine-grained sedimentary system: A case study of organic-rich shale in first member of Cretaceous Qingshankou Formation in Gulong Sag, Songliao Basin, NE China. Pet. Explor. Dev. 2018, 45 (5), 884–894. 10.1016/S1876-3804(18)30091-0.

[ref59] Xiao F.; Yang J.; Li S.; Gong F.; Zhang J.; Yao Y.; Li A.; Zhang L.; Huang Y.; Su F.; Bai Y. Geological and Geochemical characteristics of the first member of the Cretaceous Qingshankou Formation in the Qijia Sag, Northern Songliao Basin, Northeast China: Implication for its shale oil enrichment. Geofluids 2021, 2021, 9989792 10.1155/2021/9989792.

[ref60] Yang J.; Wang L.; Li S.; Zuo C.; Xiao F.; Chen Y.; Yao Y.; Bai L. The influence of reservoir composition on the pore structure of continental shale: A case study from the Qingshankou Formation in the Sanzhao Sag of Northern Songliao Basin, NE China. Geofluids 2021, 2021, 5869911 10.1155/2021/5869911.

[ref61] Yao Y.; Xiao F.; Li S.; Yang J.; Huang Y.; Ye C. Enrichment model of tight shale oil in the first Member of Cretaceous Qingshankou Formation in the southern Qijia sag, Songliao basin. Acta Geol. Sin. 2024, 98 (11), 3393–3407.

[ref62] Zhao Z.; Littke R.; Zieger L.; Hou D.; Froidl F. Depositional environment, thermal maturity and shale oil potential of the Cretaceous Qingshankou Formation in the eastern Changling Sag, Songliao Basin, China: An integrated organic and inorganic geochemistry approach. Int. J. Coal Geol. 2020, 232, 103621 10.1016/j.coal.2020.103621.

[ref63] Wu Z.; Littke R.; Baniasad A.; Yang Z.; Tang Z.; Grohmann S. Geochemistry and petrology of petroleum source rocks in the Upper Cretaceous Qingshankou Formation, Songliao Basin. NE China. Int. J. Coal Geol. 2023, 270, 104222 10.1016/j.coal.2023.104222.

[ref64] Dong T.; He S.; Yin S.; Wang D.; Hou Y.; Guo J. Geochemical characterization of source rocks and crude oils in the Upper Cretaceous Qingshankou Formation, Changling Sag, southern Songliao Basin. Mar. Pet. Geol. 2015, 64, 173–188. 10.1016/j.marpetgeo.2015.03.001.

[ref65] Xiao F.; Yang J.; Li S.; Yao Y.; Huang Y.; Gao X. Enrichment and movability of lacustrine tight shale oil for the first member of the Upper Cretaceous Qingshankou Formation in the Sanzhao Sag, Songliao Basin, NE China: Insights from saturated hydrocarbon molecules. Fuel 2024, 368, 131615 10.1016/j.fuel.2024.131615.

[ref66] Hou Q.; Feng Z. Q.; Feng Z. H. Continental petroleum geology of the Songliao basin; Pet. Industry Press: Beijing, 2009.

[ref67] Feng Z.; Jia C.; Xie X.; Zhang S.; Feng Z.; Timothy A. C. Tectono stratigraphic units and stratigraphic sequences of the nonmarine Songliao basin, northeast China. Basin Res. 2010, 22 (1), 79–95. 10.1111/j.1365-2117.2009.00445.x.

[ref68] Zhang L.; Lu S.; Wang W. Source rocks and hydrocarbon migration models of Fuyu-Yangdachengzi oil layer in the Chaoyanggou Terrace, Songliao Basin. Chin. J. Geol. 2009, 44 (2), 560–570.

[ref69] Liu F.; Wang X.; Liu Z.; Tian F.; Zhao Y.; Pan G.; Peng C.; Liu T.; Zhao L.; Zhang K.; Zhang S.; Liu X.; Zhao R. Identification of tight sandstone reservoir lithofacies based on CNN image recognition technology: A case study of Fuyu reservoir of Sanzhao Sag in Songliao Basin. Geoenergy Sci. Eng. 2023, 222, 211459 10.1016/j.geoen.2023.211459.

[ref70] Lin R.; Wang P.; Dai Y.; Zhang Z.; Huang G.; Bao J. Petroleum geochemical significance of PAH in fossil fuel. In Geol. Soc. China, Pet. Soc. China and Chin. Soc. for Mineral., Petrol. and Geochem. Collection on Organic Geochem; Geol. Publ. House: Beijing, 1987; pp 129–140.

[ref71] Hatcher P. G. Chemical structural models for coalified wood (vitrinite) in low rank coal. Org. Geochem. 1990, 16 (4/5/6), 959–968. 10.1016/0146-6380(90)90132-J.

[ref72] Tuo J. Geochemistry of the Tertiary aromatic hydrocarbons in the Qaidam Basin——Relationship between dicyclic and polycyclic aromatic hydrocarbons. Exp. Pet. Geol. 1996, 18 (4), 406–412.

[ref73] Grice K.; Nabbefeld B.; Maslen E. Source and significance of selected polycyclic aromatic hydrocarbons in sediments (Hovea-3 well, Perth Basin, Western Australia) spanning the Permian–Triassic boundary. Org. Geochem. 2007, 38, 1795–1803. 10.1016/j.orggeochem.2007.07.001.

[ref74] Xia Y.; Wang C.; Meng Q.; Wang H.; Du L. Formation mechanism of biphenyl series and benzothiophene series. Sci. China 1999, 29 (3), 257–262.

[ref75] Laflamme R. E.; Hites R. A. The global distribution of polycyclic aromatic hydrocarbons in recent sediments. Geochim. Cosmochim. Acta 1978, 42, 289–303. 10.1016/0016-7037(78)90182-5.

[ref76] Tan Y.; Heit M. Biogenic and abiogenic polynuclear aromatic hydrocarbons in sediments from two remote Adirondack lakes. Geochim. Cosmochim. Acta 1981, 45, 2267–2279. 10.1016/0016-7037(81)90076-4.

[ref77] Jiang N.; Tong Z.; Ren D.; Song F.; Yang D. The discovery of retene in Precambrian and Lower Paleozoic marine formations. Chin. J. Geochem. 1995, 14 (1), 41–51. 10.1007/BF02840382.

[ref78] Zhang L.; Huang D.; Liao Z. High concentration retene and methylretene in Silurian carbonate of Michigan Basin. Chin. Sci. Bull. 1999, 44 (22), 2083–2086. 10.1007/BF02884927.

[ref79] Zhou W.; Wang R.; Radke M.; Wu Q.; Sheng G.; Liu Z. Retene in pyrolysates of algal and bacterial organic matter. Org. Geochem. 2000, 31 (7/8), 757–762. 10.1016/S0146-6380(00)00064-4.

[ref80] Mo S.; Tang Y.; Zhang J.; He D.; Su F.; Sun L. Geochemical characteristics of aromatics in source rocks of the Upper Cretaceous Nenjiang Formation in the Well JTD1 from the Western Slope Area in the Songliao Basin. J. Oil Gas Technol. 2020, 42 (2), 46–55. 10.12677/JOGT.2020.422015.

[ref81] Killops S. D.; Massoud M. S. Polycyclic aromatic hydrocarbons of pyrolytic origin in ancient sediments: evidence for Jurassic vegetation fires. Org. Geochem. 1992, 18, 1–7. 10.1016/0146-6380(92)90137-M.

[ref82] Li M.; Shi S.; Wang T.-G. Identification and distribution of chrysene, methylchrysenes and their isomers in crude oils and rock extracts. Org. Geochem. 2012, 52, 55–66. 10.1016/j.orggeochem.2012.08.011.

[ref83] Fang R.; Li M.; Wang T.-G.; Zhang L.; Shi S. Identification and distribution of pyrene, methylpyrenes and their isomers in rock extracts and crude oils. Org. Geochem. 2015, 83–84, 65–76. 10.1016/j.orggeochem.2015.03.003.

[ref84] Yunker M. B.; Macdonald R. W.; Vingarzan R.; Mitchell R. H.; Goyette D.; Sylvestre S. PAHs in the Fraser River basin: a critical appraisal of PAH ratios as indicators of PAH source and composition. Org. Geochem. 2002, 33, 489–515. 10.1016/S0146-6380(02)00002-5.

[ref85] Yunker M. B.; Macdonald R. W.; Snowdon L. R.; Fowler B. R. Alkane and PAH biomarkers as tracers of terrigenous organic carbon in Arctic Ocean sediments. Org. Geochem. 2011, 42, 1109–1146. 10.1016/j.orggeochem.2011.06.007.

[ref86] Luo Q.; Gong L.; Qu Y.; Zhang K.; Zhang G.; Wang S. The tight oil potential of the Lucaogou Formation from the southern Junggar Basin. China. Fuel 2018, 234, 858–871. 10.1016/j.fuel.2018.07.002.

[ref87] Wang G.; Chang X.; Wang T.-G.; Simoneit B. R. T. Pregnanes as molecular indicators for depositional environments of sediments and petroleum source rocks. Org. Geochem. 2015, 78, 110–120. 10.1016/j.orggeochem.2014.11.004.

[ref88] Noble R. A.; Alexander R.; Kagi R. I.; Knox J. Identification of some diterpenoid hydrocarbons in petroleum. Org. Geochem. 1986, 10, 825–829. 10.1016/S0146-6380(86)80019-5.

[ref89] Philp R. P.; Gilbert T. D. Biomarker distributions in Australian oils predominantly derived from terrigenous source material. Org. Geochem. 1986, 10, 73–84. 10.1016/0146-6380(86)90010-0.

[ref90] Larter S. R.; Bowler B. F. J.; Li M.; Chen M.; Brincat D.; Bennett B.; Noke K.; Donohoe P.; Simmons D.; Kohnen M.; Allan J.; Telnaes N.; Horstad I. Molecular indicators of secondary oil migration distances. Nature 1996, 383, 593–597. 10.1038/383593a0.

[ref91] Huang H.; Ren F.; Larter S. R. Effect of biodegradation on the distribution of benzocarbazole in crude oil. Sci. Bull. 2002, 47 (16), 1271–1275.

[ref92] Ghosh S.; Dutta S.; Bhattacharyya S.; Konar R.; Priya T. Paradigms of biomarker and PAH distributions in lower Gondwana bituminous coal lithotypes. Int. J. Coal Geol. 2022, 260, 104067 10.1016/j.coal.2022.104067.

