# Supplementary material for: Characterization of Polycyclic Aromatic Hydrocarbons in a Shale Strata Profile from the First Member of the Upper Cretaceous Qingshankou Formation in the Sanzhao Sag, Songliao Basin, NE China
Source: ACS Omega. 2025 Feb 13;10(7):7220–41. doi: 10.1021/acsomega.4c10835 (PMC11866183; doi:10.1021/acsomega.4c10835)
Supplement: Supplementary file 1 — ao4c10835_si_001.pdf [file ao4c10835_si_001.pdf]

# - Supporting Information –

Characterization of Polycyclic Aromatic Hydrocarbons in a Shale Strata Profile from the First Member of the Upper Cretaceous Qingshankou Formation in the Sanzhao Sag, Songliao Basin, NE China

Fei Xiao [a, b, *], *Jianguo Yang* [a, b], *Yulai Yao* [a, b, *], *Shichao Li* [a, b], *Yiming Huang* [a, b], *Xiaoyong Gao* [a, b]

[a] Shenyang Center of China Geological Survey/Northeast Geological S&T Innovation Center of China Geological Survey, Shenyang 110034, Liaoning Province, China

[b] Shale Oil Technology Innovation Center of China Geological Survey, Shenyang 110034, Liaoning Province, China

* Corresponding authors:

E-mail address:

xiaof@mail.cgs.gov.cn, 365354120@qq.com (F. Xiao); yaoyulai@mail.cgs.gov.cn (Y. Yao).

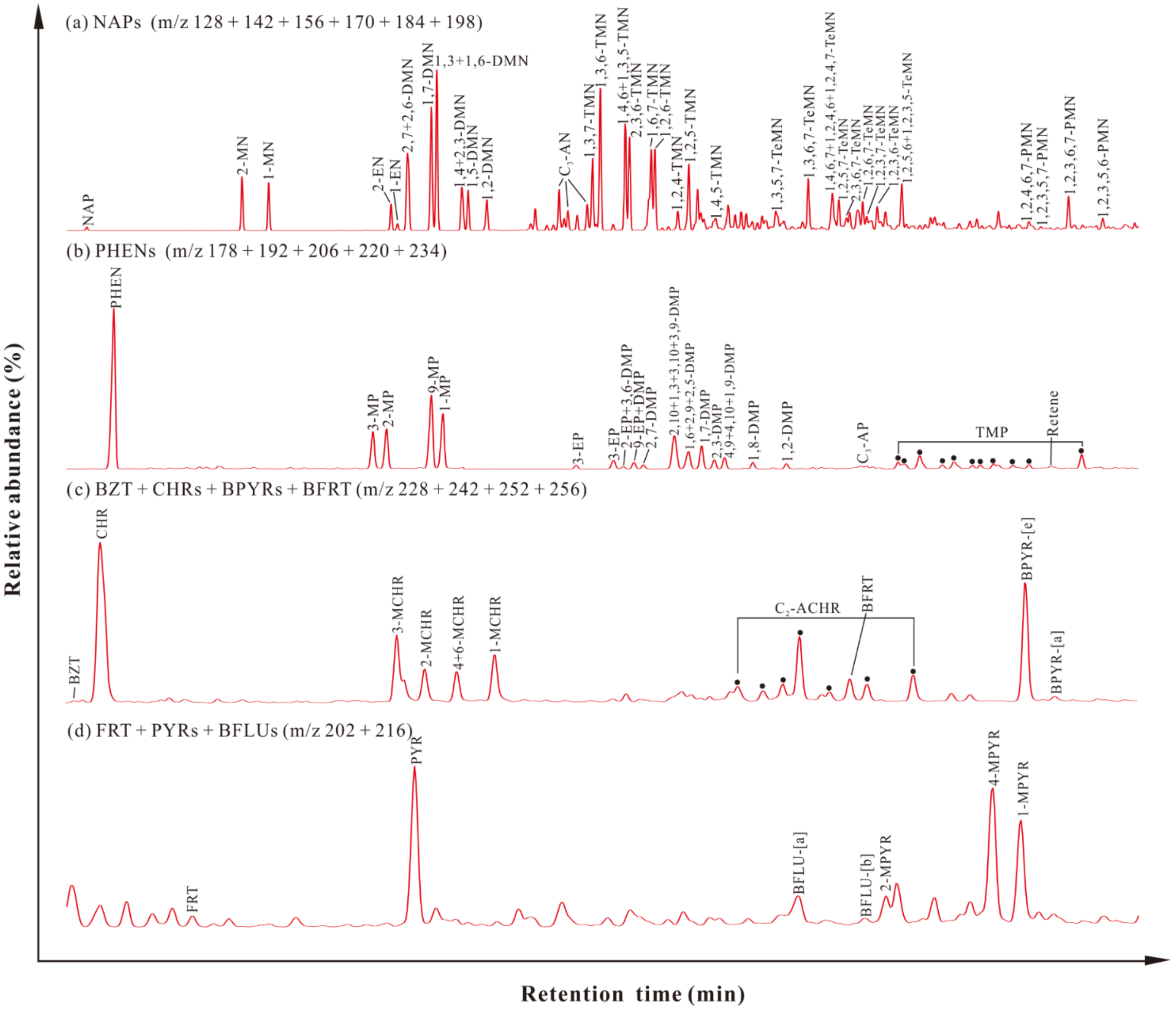

Figure S1. Mass chromatograms of the PAHs including (a) naphthalene series (NAPs), (b) phenanthrene series (PHENs), (c) benzo[a]anthracene (BZT) + chrysene series (CHRs) + benzopyrene series (BPYRs) + benzofluoranthene (BFRT), and (d) fluoranthene (FRT) + pyrene series (PYRs) + benzofluorene series (BFLUs) of the K_2qn^1 sample at the depth of 1971.04 m from the SYY3 well in the Sanzhao Sag. MN = methylnaphthalene; EN = ethylnaphthalene; DMN = dimethylnaphthalene; AN = alkylnaphthalene; TMN = trimethylnaphthalene; TeMN = tetramethylnaphthalene; PMN = pentamethylnaphthalene; MP = methylphenanthrene; EP = ethylphenanthrene; DMP = dimethylphenanthrene; AP = alkylphenanthrene; TMP = trimethylphenanthrene; CHR = chrysene; MCHR = methylchrysene; ACHR = alkylchrysene; BPYR-[e] = benzo[e]pyrene; BPYR-[a] = benzo[a]pyrene; PYR = pyrene; BFLU-[a] = benzo[a]fluorene; BFLU-[b] = benzo[b]fluorene; MPYR = methylpyrene.

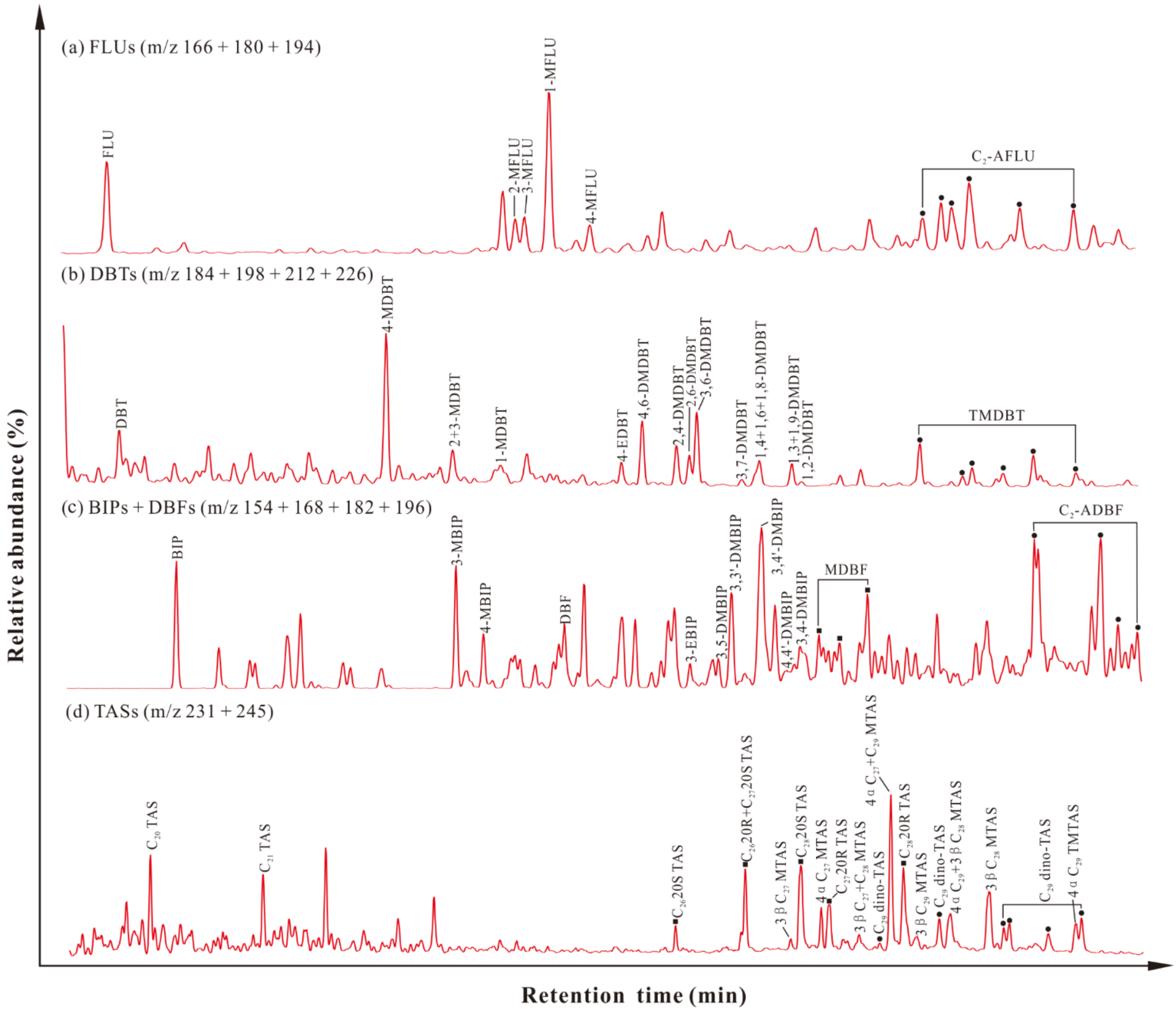

Figure S2. Mass chromatograms of the PAHs including (a) fluorene series (FLUs), (b) dibenzothiophen series (DBTs), (c) biphenyl series (BIPs) + dibenzofuran series (DBFs), and (d) triaromatic steroid series (TASs) of the K_2qn^1 sample at the depth of 1971.04 m from the SYY3 well in the Sanzhao Sag. FLU = fluorene; MFLU = methylfluorene; AFLU = alkylfluorene; DBT = dibenzothiophene; MDBT = methyldibenzothiophene; EDBT = ethyldibenzothiophene; DMDBT = dimethyldibenzothiophene; TMDBT = trimethyldibenzothiophene; BIP = biphenyl; MBIP = methylbiphenyl; DMBIP = dimethylbiphenyl; DBF = dibenzofuran; MDBF = methyldibenzofuran; ADBF = alkyldibenzofuran; TAS = triaromatic steroid; MTAS = methyl triaromatic steroid; dino-TAS = triaromatic dinosterane.

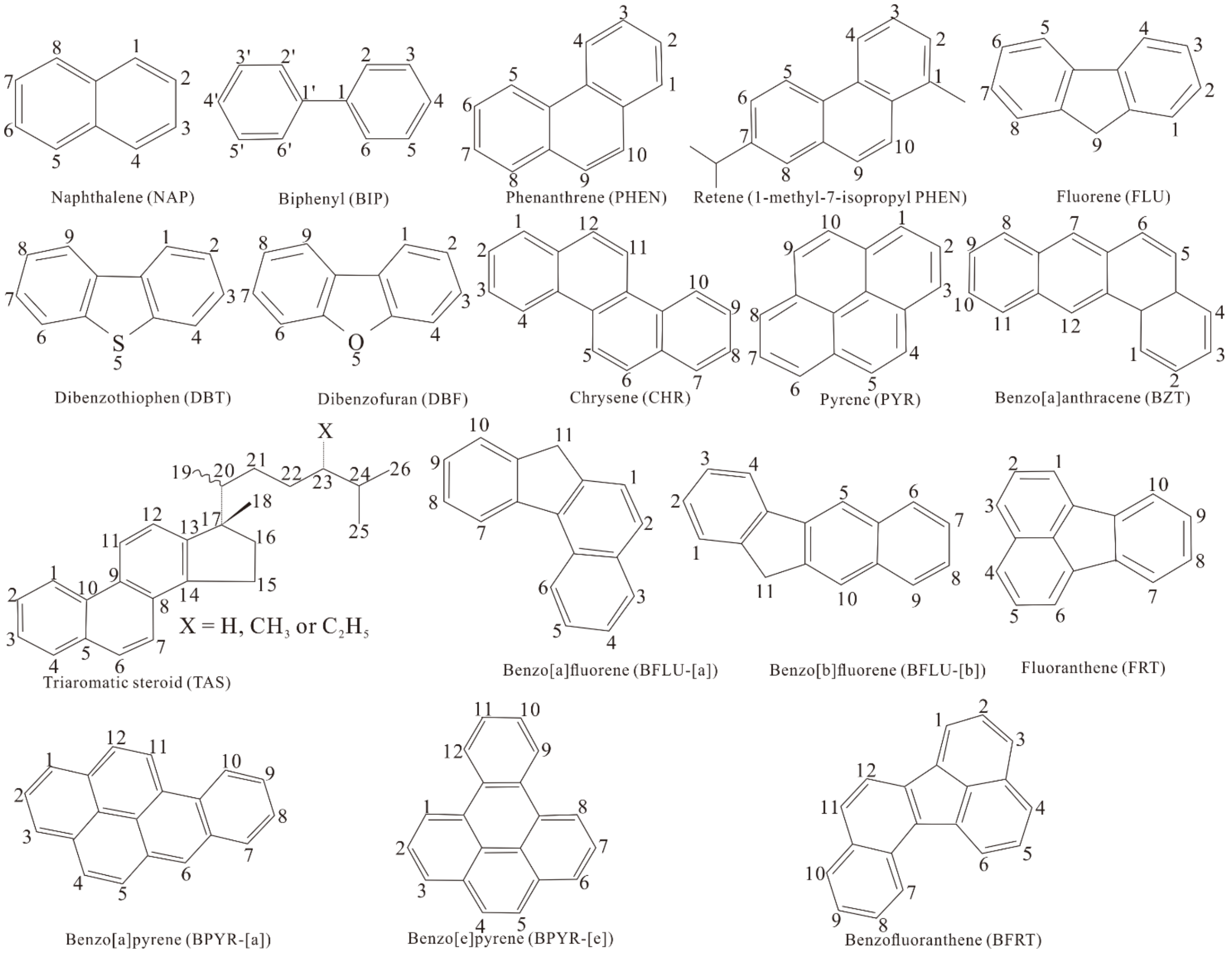

Figure S3. Basic molecular structure diagrams of major PAHs in the K_2qn^1 samples from the SYY 3 well, Sanzhao Sag

Table S1. Relative Percentage Contents of the Major PAHs Excluding NAPs and BIPs of the K_2qn^1 Samples in the SYY3 Well [a]

depth (m)	lithology	bicyclic aromatic compounds (%)		tricyclic aromatic compounds (%)				tetracyclic aromatic compounds (%)						pentacyclic aromatic compounds (%)	
		NAPs	BIPs	PHENs	FLUs	DBTs	DBFs	CHRs	PYRs	BZT	TASs	BFLUs	FRT	BPYRs	BFRT
1971.04	shale	/	/	71.15	4.08	2.74	0.91	8.96	4.53	0.05	3.56	0.49	0.14	2.93	0.46
1974.15	shale	/	/	68.05	6.88	3.06	0.87	8.90	4.58	0.05	3.46	0.58	0.13	2.93	0.52
1976.05	mudstone	/	/	69.80	5.47	2.54	0.89	8.60	4.13	0.07	4.90	0.57	0.13	2.40	0.49
1977.56	silty mudstone	/	/	69.99	4.32	2.46	0.84	9.27	4.27	0.06	4.96	0.58	0.14	2.57	0.53
1980.30	shale	/	/	66.61	8.00	3.09	0.88	8.90	4.42	0.05	4.33	0.66	0.13	2.42	0.52
1983.29	mudstone	/	/	69.84	5.62	3.22	0.98	10.28	4.22	0.08	2.49	0.49	0.11	2.27	0.41
1985.35	mudstone	/	/	69.70	4.57	3.43	0.81	10.85	4.59	0.08	2.57	0.47	0.11	2.39	0.43
1987.80	mudstone	/	/	68.83	4.95	3.51	0.84	11.30	4.60	0.07	2.58	0.47	0.10	2.32	0.44
1990.01	silty mudstone	/	/	68.36	5.17	3.13	0.86	11.03	4.21	0.07	4.10	0.47	0.09	2.09	0.40
1992.25	mudstone	/	/	69.97	5.48	3.38	0.93	10.60	4.32	0.07	2.36	0.49	0.09	1.93	0.39
1994.33	mudstone	/	/	70.40	5.69	3.49	1.02	10.16	4.25	0.06	2.28	0.48	0.12	1.69	0.37
1995.96	mudstone	/	/	69.98	5.63	3.59	1.04	10.72	4.54	0.06	1.53	0.49	0.09	1.95	0.39
1998.55	shale	/	/	71.87	5.38	3.61	1.02	10.06	4.51	0.06	0.61	0.47	0.17	1.86	0.38
2001.15	shale	/	/	70.96	5.86	3.33	0.99	10.51	4.76	0.07	0.73	0.47	0.08	1.86	0.38
2003.08	shale	/	/	70.91	5.27	3.11	0.84	11.15	4.76	0.08	0.89	0.57	0.09	1.91	0.41
2005.23	mudstone	/	/	71.50	5.23	2.92	0.82	11.10	4.67	0.08	0.79	0.52	0.10	1.87	0.41
2007.90	mudstone	/	/	71.36	5.09	3.45	0.87	10.71	5.05	0.06	0.64	0.50	0.09	1.79	0.39
2009.53	mudstone	/	/	71.08	5.34	3.50	0.81	10.93	5.14	0.07	0.27	0.42	0.08	1.99	0.38
2011.79	shale	/	/	71.29	6.06	3.45	0.88	10.39	4.93	0.05	0.27	0.48	0.06	1.82	0.34
2014.26	mudstone	/	/	72.37	5.66	3.50	0.81	9.76	4.98	0.05	0.31	0.44	0.07	1.73	0.31
2016.38	mudstone	/	/	72.58	5.22	3.67	0.84	9.76	5.10	0.06	0.28	0.38	0.08	1.74	0.31
2018.39	mudstone	/	/	72.44	6.16	3.47	0.90	9.81	4.60	0.06	0.17	0.44	0.07	1.61	0.28
2022.34	mudstone	/	/	72.55	5.96	3.67	0.88	9.78	4.71	0.06	0.14	0.43	0.07	1.50	0.25
2027.62	shale	/	/	72.30	6.49	3.60	1.03	9.71	4.37	0.07	0.23	0.45	0.06	1.45	0.25
2029.61	mudstone	/	/	72.73	6.71	3.75	1.03	9.21	4.28	0.05	0.21	0.44	0.06	1.29	0.23
2033.82	mudstone	/	/	72.20	6.64	4.31	1.01	9.61	4.06	0.07	0.28	0.41	0.06	1.13	0.21
2035.80	oil shale	/	/	69.10	5.80	4.18	0.90	12.06	4.21	0.08	0.58	0.37	0.06	2.31	0.35
2038.37	mudstone	/	/	70.15	6.34	4.29	1.03	10.94	3.98	0.05	0.58	0.39	0.06	1.90	0.30
2040.94	mudstone	/	/	69.84	6.84	4.14	1.15	10.86	3.78	0.05	0.72	0.41	0.05	1.86	0.30
2044.57	oil shale	/	/	69.28	5.84	4.45	0.95	11.66	4.25	0.07	0.61	0.39	0.05	2.13	0.32
2048.60	mudstone	/	/	69.56	5.66	4.12	0.82	12.25	3.98	0.09	0.70	0.41	0.05	2.05	0.33
2054.63	mudstone	/	/	70.32	4.41	4.04	0.75	12.98	4.08	0.11	0.46	0.32	0.06	2.15	0.33
2057.32	oil shale	/	/	69.70	6.13	4.50	0.89	11.86	3.86	0.09	0.39	0.45	0.05	1.78	0.30
2060.00	mudstone	/	/	69.08	5.97	4.52	0.95	12.84	3.48	0.10	0.13	0.49	0.09	1.98	0.37

[a]Note: / = Data are not available.

Table S2. Common Aromatic Geochemical Indexes and Typical Saturated Geochemical Indexes Indicating Biotic Input, Sedimentary Environment, and Thermal Maturity of the K_2qn^1 Samples in the SYY3 Well [a]

depth (m)	lithology	biotic input indexes					sedimentary environment indexes							thermal maturity indexes						
		1,2,5-TMN/NAPs (%)	(1,2,5,6+1,2,3,5-TeMN)/TeMNs (%)	Retene/PHENs (%)	BZT/(BZT+CHR)	FRT/(FRT+PYR)	DBFs/(DBFs+FLUs)	DBTs/(DBTs+FLUs)	DBTs/DBFs	DBT/PHEN	TAS $C_{26}20S/C_{28}20S$	Pr/Ph*	GR/C_{30}H*	*TMNr*	*TeMNr*	*PMNr*	*MPI1*	*MPI2*	*MPR*	R_o (%)*
1971.04	shale	3.48	18.77	0.28	0.071	0.014	0.18	0.40	3.01	0.013	0.20	0.80	0.44	0.52	0.51	0.42	0.36	0.37	0.65	0.84
1974.15	shale	2.89	18.36	0.28	0.064	0.016	0.11	0.31	3.52	0.015	0.20	1.44	0.42	0.54	0.52	0.43	0.38	0.38	0.70	0.83
1976.05	mudstone	3.27	17.90	0.37	0.073	0.023	0.14	0.32	2.85	0.013	0.20	1.25	0.38	0.55	0.53	0.43	0.39	0.39	0.69	0.87
1977.56	silty mudstone	3.12	17.78	0.58	0.074	0.019	0.16	0.36	2.92	0.012	0.19	1.25	0.39	0.55	0.53	0.43	0.39	0.39	0.69	0.86
1980.30	shale	2.57	17.27	0.60	0.068	0.017	0.10	0.28	3.52	0.016	0.19	1.23	0.42	0.58	0.54	0.44	0.37	0.38	0.69	0.88
1983.29	mudstone	3.75	17.19	0.35	0.064	0.018	0.15	0.36	3.27	0.014	0.21	0.89	0.37	0.53	0.53	0.49	0.38	0.39	0.72	0.84
1985.35	mudstone	5.86	18.25	0.18	0.060	0.019	0.15	0.43	4.25	0.014	0.22	1.07	0.42	0.43	0.51	0.47	0.39	0.40	0.72	0.89
1987.80	mudstone	5.41	17.64	0.37	0.056	0.015	0.14	0.41	4.18	0.016	0.21	0.83	0.65	0.47	0.52	0.49	0.39	0.40	0.72	0.85
1990.01	silty mudstone	4.80	16.75	0.25	0.059	0.015	0.14	0.38	3.63	0.011	0.21	0.82	0.65	0.50	0.54	0.50	0.39	0.40	0.72	0.87
1992.25	mudstone	4.76	16.31	0.19	0.053	0.016	0.14	0.38	3.65	0.015	0.22	0.80	0.59	0.51	0.55	0.50	0.38	0.40	0.73	0.86
1994.33	mudstone	3.86	15.10	0.20	0.072	0.015	0.15	0.38	3.43	0.016	0.26	0.75	0.51	0.56	0.58	0.54	0.39	0.40	0.74	0.87
1995.96	mudstone	2.77	15.57	0.18	0.051	0.014	0.16	0.39	3.44	0.018	0.22	1.02	0.29	0.58	0.57	0.51	0.37	0.39	0.73	0.82
1998.55	shale	2.83	15.74	0.15	0.091	0.014	0.16	0.40	3.56	0.018	0.29	1.00	0.41	0.57	0.56	0.50	0.36	0.38	0.71	0.86
2001.15	shale	1.98	12.49	0.31	0.046	0.017	0.14	0.36	3.36	0.015	0.23	0.83	0.30	0.66	0.65	0.59	0.39	0.41	0.76	0.85
2003.08	shale	3.75	13.57	0.23	0.053	0.018	0.14	0.37	3.72	0.013	0.21	0.84	0.30	0.59	0.62	0.57	0.40	0.41	0.75	0.86
2005.23	mudstone	3.65	13.96	0.20	0.055	0.017	0.14	0.36	3.57	0.011	0.23	0.90	0.28	0.58	0.61	0.56	0.39	0.40	0.74	0.86
2007.90	mudstone	3.57	13.71	0.20	0.049	0.013	0.15	0.40	3.96	0.017	0.23	0.89	0.31	0.59	0.62	0.57	0.39	0.41	0.76	0.89
2009.53	mudstone	3.44	12.47	0.28	0.043	0.015	0.13	0.40	4.31	0.016	0.26	0.90	0.32	0.63	0.66	0.58	0.41	0.43	0.76	0.87
2011.79	shale	2.82	11.73	0.21	0.033	0.013	0.13	0.36	3.94	0.015	0.25	0.93	0.31	0.66	0.68	0.60	0.41	0.43	0.77	0.86
2014.26	mudstone	3.58	11.62	0.30	0.039	0.013	0.13	0.38	4.31	0.014	0.22	0.88	0.30	0.63	0.69	0.61	0.42	0.45	0.77	0.87
2016.38	mudstone	3.07	11.32	0.24	0.041	0.014	0.14	0.41	4.39	0.015	0.26	0.87	0.27	0.66	0.70	0.61	0.42	0.44	0.77	0.88
2018.39	mudstone	2.65	10.23	0.23	0.042	0.015	0.13	0.36	3.85	0.015	0.28	0.80	0.32	0.69	0.73	0.65	0.43	0.45	0.78	0.88
2022.34	mudstone	2.86	9.80	0.16	0.044	0.015	0.13	0.38	4.18	0.015	0.26	0.76	0.38	0.69	0.74	0.65	0.43	0.46	0.77	0.89
2027.62	shale	1.88	9.44	0.22	0.039	0.017	0.14	0.36	3.49	0.015	0.24	0.72	0.32	0.73	0.75	0.65	0.43	0.46	0.78	0.87
2029.61	mudstone	1.93	8.96	0.01	0.041	0.015	0.13	0.36	3.64	0.016	0.26	0.78	0.32	0.74	0.76	0.67	0.44	0.47	0.79	0.89
2033.82	mudstone	1.94	8.43	0.20	0.045	0.021	0.13	0.39	4.25	0.018	0.40	0.76	0.38	0.75	0.78	0.69	0.48	0.51	0.81	0.93
2035.80	oil shale	2.20	8.92	0.20	0.040	0.021	0.13	0.42	4.64	0.019	0.34	0.76	0.38	0.73	0.77	0.67	0.47	0.50	0.80	0.92
2038.37	mudstone	1.97	8.91	0.16	0.042	0.016	0.14	0.40	4.15	0.019	0.34	0.77	0.36	0.75	0.77	0.68	0.47	0.50	0.82	0.92
2040.94	mudstone	1.67	8.60	0.25	0.043	0.016	0.14	0.38	3.60	0.018	0.37	1.02	0.31	0.76	0.77	0.67	0.47	0.50	0.83	0.91
2044.57	oil shale	1.99	9.13	0.18	0.039	0.021	0.14	0.43	4.69	0.020	0.35	0.82	0.32	0.74	0.76	0.67	0.46	0.50	0.81	0.95
2048.60	mudstone	2.57	9.20	0.23	0.041	0.025	0.13	0.42	5.03	0.018	0.32	0.83	0.34	0.72	0.76	0.66	0.49	0.52	0.82	0.98
2054.63	mudstone	3.06	9.47	0.17	0.046	0.027	0.15	0.48	5.36	0.018	/	0.72	0.34	0.68	0.76	0.66	0.49	0.53	0.81	0.97
2057.32	oil shale	2.64	9.28	0.18	0.043	0.024	0.13	0.42	5.03	0.021	0.44	0.72	0.33	0.71	0.76	0.65	0.49	0.52	0.83	0.97
2060.00	mudstone	2.63	9.18	0.24	0.077	0.027	0.14	0.43	4.75	0.021	/	0.91	0.52	0.72	0.77	0.61	0.49	0.52	0.83	0.98

[a]Note: 1,2,5-TMN/NAPs = (1,2,5-trimethylnaphthalene) × 100/naphthalene series; (1,2,5,6 + 1,2,3,5)-TeMN/TeMNs = (1,2,5,6 + 1,2,3,5)-tetramethylnaphthalenes × 100/tetramethylnaphthalene series; Retene/PHENs = retene × 100/phenanthrene series; BZT/(BZT + CHR) = benzo[a]anthracene/(benzo[a]anthracene + chrysene); FRT/(FRT + PYR) = fluoranthene/(fluoranthene + pyrene); DBFs/(DBFs + FLUs) = dibenzofuran series/(dibenzofuran series + fluorene series); DBTs/(DBTs + FLUs) = dibenzothiophen series/(dibenzothiophen series + fluorene series); DBTs/DBFs = dibenzothiophen series/dibenzofuran series; DBT/PHEN = dibenzothiophen/phenanthrene; *TMNr* = 1,3,7-trimethylnaphthalene/(1,3,7 + 1,2,5)-trimethylnaphthalenes; *TeMNr* = 1,3,6,7-tetramethylnaphthalene/(1,3,6,7 + 1,2,5,6 + 1,2,3,5)-tetramethylnaphthalenes; *PMNr* = 1,2,4,6,7-pentamethylnaphthalene/(1,2,4,6,7 + 1,2,3,5,6)-pentamethylnaphthalenes; *MPI1* = 1.5 × (2-methylphenanthrene + 3-methylphenanthrene)/(phenanthrene + 1-methylphenanthrene + 9-methylphenanthrene); *MPI2* = 3 × (2-methylphenanthrene)/(phenanthrene + 1-methylphenanthrene + 9-methylphenanthrene); *MPR* = 2-methylphenanthrene/1-methylphenanthrene; / = data are not available; * the data of Pr/Ph (pristane/phytane), GR/C_{30}H (gammacerane/C_{30}hopane) and R_o were referenced from the published paper of Xiao et al.[65]. Reprinted (Adapted or Reprinted in part) with permission from [Xiao, F.; Yang, J.; Li, S.; Yao, Y.; Huang, Y.; Gao, X. Enrichment and movability of lacustrine tight shale oil for the first member of the Upper Cretaceous Qingshankou Formation in the Sanzhao Sag, Songliao Basin, NE China: Insights from saturated hydrocarbon molecules. Fuel 2024, 368, 131615. https://doi.org/10.1016/j.fuel.2024.1316 15.] Copyright [2024] [Elsevier].

Table S3. Content Ratios of *a*-PAHs to *p*-PAH of NAPs, BIPs, PHENs, and CHRs of the K_2qn^1 Samples in the SYY3 Well [a]

depth (m)	lithology	MNs/ NAP	DMNs/ NAP	ENs/ NAP	TMNs/ NAP	TeMNs/ NAP	PMNs/ NAP	MBIPs/BIP	DMBIPs/BIP	MPs/PHEN	(DMPs+EPs)/PHEN	TMPs/PHEN	MCHRs/CHR	C_2-alkyl CHRs/CHR
1971.04	shale	17.89	56.39	17.89	51.79	17.75	1.33	1.32	1.48	1.20	0.91	0.51	0.87	0.70
1974.15	shale	7.29	15.93	7.29	12.99	4.21	0.34	1.08	1.06	1.23	0.81	0.48	0.97	0.83
1976.05	mudstone	11.85	38.54	11.85	37.24	12.98	1.01	1.35	1.57	1.27	0.95	0.56	1.02	0.90
1977.56	silty mudstone	11.72	35.60	11.72	33.43	11.57	0.93	1.28	1.47	1.23	0.95	0.57	1.04	0.94
1980.30	shale	8.23	19.05	8.23	15.27	4.85	0.38	1.06	1.05	1.22	0.93	0.55	1.07	0.99
1983.29	mudstone	25.49	117.26	6.04	148.27	57.05	4.80	1.64	1.96	1.23	0.85	0.43	0.79	0.67
1985.35	mudstone	1.96	36.46	0.80	256.48	170.31	15.66	12.14	40.73	1.34	0.97	0.49	0.78	0.67
1987.80	mudstone	3.66	136.77	3.88	567.26	317.58	28.19	7.35	17.72	1.32	0.95	0.49	0.87	0.68
1990.01	silty mudstone	10.84	229.86	7.81	602.01	294.82	25.27	4.17	7.94	1.33	0.98	0.52	0.89	0.73
1992.25	mudstone	11.39	279.34	9.27	729.15	335.51	26.71	4.42	8.22	1.28	0.91	0.46	0.87	0.66
1994.33	mudstone	39.53	281.55	13.46	484.14	204.00	17.64	2.44	3.73	1.30	0.93	0.47	0.88	0.67
1995.96	mudstone	15.98	37.93	2.14	36.49	12.02	0.88	1.14	1.18	1.23	0.87	0.44	0.89	0.69
1998.55	shale	12.78	34.11	1.97	32.48	10.57	0.80	1.21	1.21	1.25	0.82	0.37	0.84	0.56
2001.15	shale	14.10	33.93	1.71	28.69	8.04	0.52	1.05	0.98	1.29	0.89	0.42	0.89	0.63
2003.08	shale	29.74	623.11	20.81	1246.53	489.62	35.61	3.42	5.17	1.32	0.94	0.46	0.90	0.63
2005.23	mudstone	48.95	543.50	20.63	922.53	349.14	25.75	2.55	3.59	1.28	0.89	0.43	0.88	0.62
2007.90	mudstone	77.50	1083.83	36.81	1857.38	680.98	47.64	2.75	3.77	1.30	0.90	0.43	0.89	0.61
2009.53	mudstone	19.39	425.04	11.58	928.51	342.74	21.03	3.72	5.91	1.40	0.99	0.44	0.89	0.57
2011.79	shale	57.53	563.01	19.53	838.62	274.01	16.62	2.29	2.91	1.40	1.00	0.45	0.89	0.55
2014.26	mudstone	4.66	146.82	2.84	539.44	237.07	14.57	7.82	17.74	1.50	1.10	0.50	0.89	0.52
2016.38	mudstone	37.26	370.20	10.34	811.70	311.56	17.70	3.02	5.30	1.49	1.09	0.49	0.88	0.52
2018.39	mudstone	62.87	896.14	23.39	1526.73	503.38	27.46	2.79	3.88	1.49	1.14	0.53	0.94	0.56
2022.34	mudstone	37.05	968.02	23.29	2122.92	756.86	40.78	3.96	6.65	1.55	1.20	0.56	0.96	0.59
2027.62	shale	19.06	58.86	2.17	66.63	19.07	0.94	1.38	1.57	1.52	1.20	0.57	0.97	0.60
2029.61	mudstone	34.52	121.72	3.97	145.61	41.96	2.04	1.47	1.70	1.52	1.21	0.58	0.99	0.62
2033.82	mudstone	24.42	94.28	2.94	134.65	42.49	1.89	1.64	2.29	1.72	1.52	0.78	1.17	0.84
2035.80	oil shale	23.26	93.35	2.89	155.71	51.56	2.34	1.78	2.78	1.68	1.44	0.71	1.26	1.11
2038.37	mudstone	45.98	245.35	7.36	322.64	97.06	4.71	1.64	2.18	1.64	1.38	0.67	1.22	1.07
2040.94	mudstone	9.87	33.39	1.16	36.06	9.76	0.43	1.34	1.55	1.60	1.37	0.67	1.26	1.11
2044.57	oil shale	32.40	126.28	3.43	176.02	55.29	2.56	1.51	2.16	1.67	1.45	0.71	1.26	1.12
2048.60	mudstone	16.70	193.49	4.34	473.90	187.76	9.99	3.19	6.41	1.76	1.58	0.80	1.30	1.19
2054.63	mudstone	15.37	305.19	4.82	1422.19	663.08	34.38	7.77	22.73	1.88	1.71	0.85	1.28	1.15
2057.32	oil shale	29.83	382.91	8.27	977.49	381.03	18.40	3.14	6.32	1.74	1.51	0.74	1.30	1.10
2060.00	mudstone	22.51	477.63	11.30	1249.27	499.59	28.18	3.39	6.27	1.70	1.53	0.80	1.37	1.15

[a]Note: NAP = naphthalene; MNs = methylnaphthalene series; DMNs = dimethylnaphthalene series; ENs = ethylnaphthalene series; TMNs = trimethylnaphthalene series; TeMNs = tetramethylnaphthalene series; PMNs = pentamethylnaphthalene series; BIP = biphenyl; MBIPs = methylbiphenyl series; DMBIPs = dimethylbiphenyl series; PHEN = phenanthrene; MPs = methylphenanthrene series; DMPs = dimethylphenanthrene series; EPs = ethylphenanthrene series; TMPs = trimethylphenanthrene series; CHR = chrysene; MCHRs = methylchrysene series; C_2-alkyl CHRs = C_2 alkylchrysene series.

Table S4. Content Ratios of *a*-PAHs to *p*-PAH of PYRs, FLUs, DBTs, and DBFs, and Three Main Ratios of *p*-PAH Isomers of the K_2qn^1 Samples in the SYY3 Well [a]

depth (m)	lithology	MPYRs/PYR	MFLUs/FLU	C_2-alkyl FLUs/FLU	MDBTs/DBT	DMDBTs/DBT	TMDBTs/DBT	MDBFs/DBF	DMDBFs/DBF	BFLU-[b]/BFLU-[a]	BPYR-[a]/BPYR-[e]	BZT/CHR
1971.04	shale	1.43	2.72	3.50	3.55	4.32	1.86	3.49	10.15	0.12	0.035	0.014
1974.15	shale	1.46	2.41	2.78	3.39	4.26	1.99	3.46	10.28	0.11	0.034	0.017
1976.05	mudstone	1.44	2.49	2.77	3.54	4.39	2.06	3.67	11.44	0.10	0.035	0.024
1977.56	silty mudstone	1.46	2.48	2.94	3.60	4.54	2.12	3.39	10.72	0.11	0.036	0.020
1980.30	shale	1.50	2.34	2.59	3.32	4.40	2.25	2.85	10.00	0.11	0.035	0.018
1983.29	mudstone	1.72	2.32	2.76	3.52	4.79	2.36	5.08	13.36	0.15	0.035	0.018
1985.35	mudstone	1.78	3.76	5.12	4.05	5.66	2.79	11.17	38.77	0.14	0.036	0.019
1987.80	mudstone	1.82	3.15	3.99	3.50	4.81	2.38	9.78	30.58	0.07	0.034	0.015
1990.01	silty mudstone	1.87	2.96	3.84	4.39	6.50	3.46	7.74	23.51	0.07	0.037	0.015
1992.25	mudstone	1.80	2.74	3.25	3.56	4.85	2.46	8.16	23.53	0.09	0.037	0.016
1994.33	mudstone	1.78	2.62	3.32	3.35	4.60	2.42	6.47	17.68	0.08	0.039	0.016
1995.96	mudstone	1.85	2.21	2.55	3.15	4.05	2.02	5.41	13.19	0.17	0.039	0.015
1998.55	shale	1.70	2.34	2.81	3.09	3.68	1.58	5.14	12.45	0.15	0.042	0.014
2001.15	shale	1.81	2.23	2.49	3.39	4.32	2.10	4.32	9.85	0.08	0.039	0.017
2003.08	shale	1.82	2.78	3.52	3.79	5.14	2.54	6.01	16.60	0.16	0.040	0.019
2005.23	mudstone	1.80	2.74	3.40	4.00	5.38	2.65	5.16	13.70	0.06	0.039	0.017
2007.90	mudstone	1.80	2.67	3.39	3.31	4.17	1.95	6.12	15.74	0.15	0.041	0.013
2009.53	mudstone	1.82	2.97	3.95	3.58	4.69	2.15	6.22	15.98	0.06	0.035	0.015
2011.79	shale	1.81	2.52	3.03	3.79	5.13	2.34	5.27	12.53	0.08	0.031	0.013
2014.26	mudstone	1.84	3.47	4.60	4.14	5.90	2.76	7.65	21.81	0.07	0.031	0.013
2016.38	mudstone	1.83	3.23	4.50	4.02	5.55	2.56	6.33	17.38	0.13	0.033	0.014
2018.39	mudstone	1.87	2.83	3.71	4.02	5.79	2.74	5.18	13.02	0.19	0.035	0.015
2022.34	mudstone	1.91	3.19	4.32	4.11	6.11	2.94	6.00	15.79	0.19	0.036	0.015
2027.62	shale	1.97	2.61	3.28	3.94	5.91	2.96	4.33	10.39	0.20	0.037	0.017
2029.61	mudstone	1.97	2.58	3.23	3.96	5.94	2.97	4.25	10.01	0.20	0.038	0.015
2033.82	mudstone	2.17	2.96	4.17	4.34	7.46	4.09	4.93	11.48	0.21	0.048	0.022
2035.80	oil shale	2.19	3.14	4.50	4.09	6.72	3.51	4.91	11.22	0.19	0.046	0.022
2038.37	mudstone	2.14	2.80	3.76	4.02	6.47	3.44	4.70	11.58	0.22	0.048	0.016
2040.94	mudstone	2.16	2.49	3.20	4.03	6.58	3.52	4.06	9.28	0.22	0.051	0.017
2044.57	oil shale	2.22	2.99	4.11	4.12	6.75	3.55	4.59	10.60	0.19	0.047	0.021
2048.60	mudstone	2.23	3.65	5.50	4.37	7.34	3.96	5.84	15.04	0.18	0.054	0.025
2054.63	mudstone	2.26	5.07	9.34	4.58	7.86	4.20	6.58	21.54	0.16	0.056	0.028
2057.32	oil shale	2.22	3.56	5.13	4.01	6.63	3.49	5.78	14.58	0.23	0.051	0.025
2060.00	mudstone	2.20	3.40	5.05	3.71	6.73	4.10	4.01	10.16	0.23	0.046	0.028

[a]Note: PYR = pyrene; MPYRs = methylpyrene series; FLU = fluorene; MFLUs = methylfluorene series; C_2-alkyl FLUs = C_2 alkyl fluorene series; DBT = dibenzothiophen; MDBTs = methyldibenzothiophen series; DMDBTs = dimethyldibenzothiophene series; TMDBTs = trimethyldibenzothiophene series; DBF = dibenzofuran; MDBFs = methyldibenzofuran series; DMDBFs = dimethyldibenzofuran series; BFLU-[a] = benzo[a]fluorene; BFLU-[b] = benzo[b]fluorene; BPYR-[a] = benzo[a]pyrene; BPYR-[e] = benzo[e]pyrene; BZT = benzo[a]anthracene; CHR = chrysene.
